# Brain-based measures of nociception during general anesthesia with remifentanil: A randomized controlled trial

**DOI:** 10.1371/journal.pmed.1003965

**Published:** 2022-04-22

**Authors:** Keerthana Deepti Karunakaran, Barry D. Kussman, Ke Peng, Lino Becerra, Robert Labadie, Rachel Bernier, Delany Berry, Stephen Green, David Zurakowski, Mark E. Alexander, David Borsook

**Affiliations:** 1 The Center for Pain and the Brain, Department of Anesthesiology, Critical Care and Pain Medicine, Boston Children’s Hospital, Harvard Medical School, Boston, Massachusetts, United States of America; 2 Division of Cardiac Anesthesia, Department of Anesthesiology, Critical Care and Pain Medicine, Boston Children’s Hospital, Harvard Medical School, Boston, Massachusetts, United States of America; 3 Département en Neuroscience, Centre de Recherche du CHUM, l’Université de Montréal Montreal, Québec, Canada; 4 Division of Biostatistics, Department of Anesthesiology, Critical Care and Pain Medicine, Boston Children’s Hospital, Harvard Medical School, Boston, Massachusetts, United States of America; 5 Department of Cardiology, Boston Children’s Hospital, Harvard Medical School, Boston, Massachusetts, United States of America; 6 Department of Psychiatry and Radiology, Massachusetts General Hospital, Boston, Massachusetts, United States of America; Erasme University Hospital, BELGIUM

## Abstract

**Background:**

Catheter radiofrequency (RF) ablation for cardiac arrhythmias is a painful procedure. Prior work using functional near-infrared spectroscopy (fNIRS) in patients under general anesthesia has indicated that ablation results in activity in pain-related cortical regions, presumably due to inadequate blockade of afferent nociceptors originating within the cardiac system. Having an objective brain-based measure for nociception and analgesia may in the future allow for enhanced analgesic control during surgical procedures. Hence, the primary aim of this study is to demonstrate that the administration of remifentanil, an opioid widely used during surgery, can attenuate the fNIRS cortical responses to cardiac ablation.

**Methods and findings:**

We investigated the effects of continuous remifentanil on cortical hemodynamics during cardiac ablation under anesthesia. In a randomized, double-blinded, placebo (PL)-controlled trial, we examined 32 pediatric patients (mean age of 15.8 years,16 females) undergoing catheter ablation for cardiac arrhythmias at the Cardiology Department of Boston Children’s Hospital from October 2016 to March 2020; 9 received 0.9% NaCl, 12 received low-dose (LD) remifentanil (0.25 mcg/kg/min), and 11 received high-dose (HD) remifentanil (0.5 mcg/kg/min). The hemodynamic changes of primary somatosensory and prefrontal cortices were recorded during surgery using a continuous wave fNIRS system. The primary outcome measures were the changes in oxyhemoglobin concentration (Nadir_HbO_, i.e., lowest oxyhemoglobin concentration and Peak_HbO_, i.e., peak change and area under the curve) of medial frontopolar cortex (mFPC), lateral prefrontal cortex (lPFC) and primary somatosensory cortex (S1) to ablation in PL versus remifentanil groups. Secondary measures included the fNIRS response to an auditory control condition. The data analysis was performed on an intention-to-treat (ITT) basis. Remifentanil group (dosage subgroups combined) was compared with PL, and a post hoc analysis was performed to identify dose effects. There were no adverse events. The groups were comparable in age, sex, and number of ablations. Results comparing remifentanil versus PL show that PL group exhibit greater Nadir_HbO_ in inferior mFPC (mean difference (MD) = 1.229, 95% confidence interval [CI] = 0.334, 2.124, *p* < 0.001) and superior mFPC (MD = 1.206, 95% CI = 0.303, 2.109, *p* = 0.001) and greater Peak_HbO_ in inferior mFPC (MD = −1.138, 95% CI = −2.062, −0.214, *p* = 0.002) and superior mFPC (MD = −0.999, 95% CI = −1.961, −0.036, *p* = 0.008) in response to ablation. S1 activation from ablation was greatest in PL, then LD, and HD groups, but failed to reach significance, whereas lPFC activation to ablation was similar in all groups. Ablation versus auditory stimuli resulted in higher Peak_HbO_ in inferior mFPC (MD = 0.053, 95% CI = 0.004, 0.101, *p* = 0.004) and superior mFPC (MD = 0.052, 95% CI = 0.013, 0.091, *p* < 0.001) and higher Nadir_HbO_ in posterior superior S1 (Pos. SS1; MD = −0.342, 95% CI = −0.680, −0.004, *p* = 0.007) during ablation of all patients. Remifentanil group had smaller Nadir_HbO_ in inferior mFPC (MD = 0.098, 95% CI = 0.009, 0.130, *p* = 0.003) and superior mFPC (MD = 0.096, 95% CI = 0.008, 0.116, *p* = 0.003) and smaller Peak_HbO_ in superior mFPC (MD = −0.092, 95% CI = −0.680, −0.004, *p* = 0.007) during both the stimuli. Study limitations were small sample size, motion from surgery, indirect measure of nociception, and shallow penetration depth of fNIRS only allowing access to superficial cortical layers.

**Conclusions:**

We observed cortical activity related to nociception during cardiac ablation under general anesthesia with remifentanil. It highlights the potential of fNIRS to provide an objective pain measure in unconscious patients, where cortical-based measures may be more accurate than current evaluation methods. Future research may expand on this application to produce a real-time indication of pain that will aid clinicians in providing immediate and adequate pain treatment.

**Trial registration:**

ClinicalTrials.gov NCT02703090

## Abstract 2

In a randomized controlled trial, Keerthana Karunakaran, Barry Kussman, and colleagues study whether remifentanil attenuates pain-related brain activity during cardiac ablation surgery.

## Author summary

**Why was this study done?:**

- It is unclear whether general anesthesia during surgery blocks activation of pain from reaching the brain.
- Most general anesthetic drugs do not produce analgesia.
- Postoperative chronic pain continues to be an enormous health problem; having an objective marker of pain and analgesia during surgery may allow anesthesiologists to better treat pain during surgery and potentially prevent the long-term complications.
- We used a brain imaging technique called functional near-infrared spectroscopy (fNIRS) to assess analgesic efficacy. Specifically, we examined the effects of an opioid called remifentanil on brain activity in patients undergoing treatment for arrythmias that require lesioning of heart nerve bundles that produce abnormal cardiac rhythms.

**What did the researchers do and find?:**

- Patients who received remifentanil when compared to those who received placebo (PL) had a decrease in brain activity during painful surgical procedure (i.e., catheter ablation).
- Dose of the remifentanil may affect the brain response to painful surgical procedure (ablation).
- Remifentanil also decreased the brain responses of patients under general anesthesia to sounds, i.e., suppressed activity in pain-related brain regions during nonpainful stimuli.

**What do these findings mean?:**

- Currently, there are no objective measures of pain. fNIRS may provide such measures in conscious and unconscious states and may be applicable to all patients undergoing general anesthesia for surgery.
- Given that surgically induced pain produces chronic pain in over 30% of patients, control of pain while in the unconscious state produced by general anesthesia may limit postoperative pain medication requirements and eliminate chronic postoperative pain.
- In future, the use of brain imaging-based markers such as fNIRS may provide the ability to immediately determine, evaluate, and block pain signals produced by surgery; the approach may also be considered for evaluating treatment efficacy in chronic pain patients.

## Introduction

General anesthesia is a reversible drug-induced state characterized by unconsciousness, amnesia, analgesia, and immobility [1]. Surgery results in nociceptor activation, inflammation at the surgical site, and nerve injury [2], thereby triggering central sensitization [3]. However, there are challenges in providing complete and consistent analgesia during the intra- and postoperative period. Opioid analgesics are the mainstay of multimodal general anesthesia for the management of nociception intraoperatively and pain postoperatively [4], but the dosage, timing of administration, and efficacy in preventing nociceptive activity from reaching the brain is not well understood.

Opioids may contribute to analgesia through peripheral effects on inflammation and pain [5–7] and, centrally, by enhancing mechanisms that include blockade of spinal synaptic transmission [8] and activation of descending analgesic pathways (e.g., periaqueductal gray) [9]. Opioids that act on mu receptors may block synaptic transmission to limit stimuli along pathways that reach thalamic and cortical regions. The magnitude of this blockade may depend on the dose of the opioid drug. However, the dosage of opioid is variable because the intraoperative administration of opioids by anesthesiologists is guided by patient weight, age, autonomic responses (changes in heart rate, blood pressure, and pupillary size) via the nociceptive medullary autonomic circuit [10], and impact on hemodynamic stability. In other words, indirect measures of analgesia are used during general anesthesia to guide intraoperative control of pain [11].

The development of technologies to provide real-time objective measures of both pain and analgesia using brain responses would allow detection of evoked (i.e., from surgery) and ongoing pain to provide an overall quantitation of “pain load” to enable appropriate pain control. Adequate pain management during surgery reduces the risk of severe acute postoperative pain, which could also reduce the unnecessary dependence on high-dose (HD) opioids and, subsequently, unwanted opioid-related side effects [12–14]. The use of brain-based markers could provide the opportunity to either administer appropriate analgesics during surgery or better postoperative pain management to minimize central sensitization. Neuroimaging technology that has revolutionized our understanding of the central nervous system in pain processing is yet to translate into a significant clinical utility due to limitations in feasibility, sensitivity, and specificity of imaging-based pain measures. Our group has demonstrated the use of functional near-infrared spectroscopy (fNIRS) to measure the cortical correlates of both evoked pain [15,16] and the effects of morphine in diminishing evoked pain response [17]. fNIRS is a promising candidate for intraoperative nociceptive monitoring due to its relatively low cost, portability, ease of use, and feasibility in real-world settings without compromising on the temporal and spatial resolution of the cortical measures. We have previously reported cortical measures of nociception in patients under general anesthesia [18].

In this present study, we wished to extend our prior fNIRS findings in patients undergoing catheter ablation of cardiac arrhythmias under general anesthesia by evaluating the effects of remifentanil in a randomized, double-blinded, placebo (PL)-controlled trial. Remifentanil, a mu-opioid receptor agonist, is a fentanyl derivative that is ultra-short acting and used as part of the induction and maintenance of general anesthesia [19]. Previous fNIRS measures in awake, anesthetized, and analgesic studies focused on activation in the somatosensory cortex (S1) and in medial frontopolar cortex (mFPC), which respond in opposite directions (anticorrelated) to evoked nociception/pain but are both attenuated by analgesia [15,17,18,20]. mFPC is activated across multiple imaging (functional magnetic resonance imaging [fMRI], positron emission tomography, and fNIRS) pain studies and likely represents a higher-order evaluative/cognitive area that has connections to multiple brain regions [21]. S1, on the other hand, is well known to be involved in the sensory discriminant aspect of nociceptive processing. We hypothesized that a continuous infusion of remifentanil in patients under general anesthesia with a volatile agent would decrease or reverse the nociceptive responses in S1 and mFPC compared to PL and that a higher dose of remifentanil would have a greater effect.

Additionally, while opioids may affect pain processing and induce abnormal pain sensitivity [22,23], they are not known to affect other sensory systems, such as auditory processing at normal doses. Responses to tones and other noises occur in marmoset monkeys under opioid anesthesia [24], although higher doses may produce sensorineural hearing loss [25]. fMRI studies in humans under general anesthesia have found that the primary (Heschl’s gyrus) and association auditory cortices remain responsive to auditory stimuli [26,27]. Hence, we used a control condition presenting auditory stimuli instructing the patients to perform a motor imagery task while under anesthesia. We hypothesized that there would be no differences observed in the fNIRS measures to auditory stimuli across the 3 conditions (PL, LD, or HD remifentanil), further supporting CNS processing of sensory stimuli under anesthesia and that the opioid effects observed for painful stimuli are consistent with the well-known analgesic effects of the drugs.

## Materials and methods

### Participants

**Fig 1:**
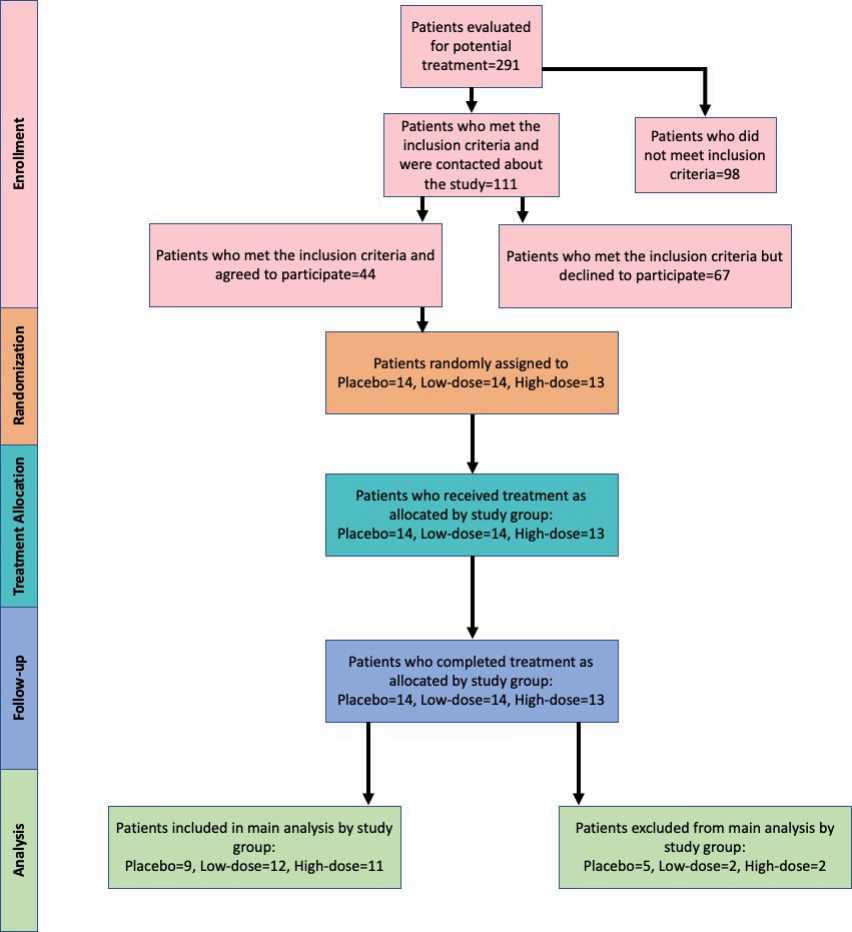
CONSORT participant flow diagram. CONSORT, Consolidated Standards of Reporting Trials.

This study is reported as per the Consolidated Standards of Reporting Trials (CONSORT) guideline (S1 Checklist). A CONSORT and SPIRIT Extension for Randomized Clinical Trials in Extenuating Circumstances (CONSERVE) checklist (S2 Checklist) is also provided to report the modifications in the trial caused due to the coronavirus pandemic. The details of participants evaluated for the study (identified, screened, randomized, and analyzed on an intention-to-treat (ITT) basis) are summarized in the flow diagram (**Fig 1**). Patients were recruited through the normal caseload of Cardiac Surgery at Boston Children’s Hospital. The research team contacted all patients scheduled to undergo elective electrophysiology study with catheter ablation of an arrhythmia under general anesthesia via email. Interested patients were screened and evaluated for eligibility before the preoperative appointment over the phone. A total of 41 patients were enrolled from October 2016 to March 2020; the number of patients is lower than the intended sample size due to the coronavirus pandemic in the United States of America, as all data collection had to be halted in March 2020. Eligible participants were 12 to 30 years of age with a structurally normal heart (by echocardiography), right handed, American Society of Anesthesiology Physical Status I or II, and spoke English. Exclusion criteria included left-handedness, smoking, structural heart disease, and a significant medical history (i.e., neurological or muscular disease, diabetes mellitus, or other syndromes of greater than minor severity). Additionally, individuals whose scalp hair did not allow sufficient optical detection or were unable to understand the study were excluded. Prior to enrollment, on the day of the surgery, the aims of the study and participation requirements were explained to the patient. If the patient was under 18 years of age, the procedure was explained to the guardian, usually the parent. Written informed consent was obtained from the participants or their respective parents/guardians before the study. Written assent was obtained from children above 7 years of age. All study procedures were approved by the Institutional Review Board (IRB-P00021030) of Boston Children’s Hospital, Boston, Massachusetts, and the study is registered with ClinicalTrials.gov (NCT02703090). A copy of the trial protocol is provided in the S1 Protocol.

### Randomization, allocation concealment, and blinding

Using a parallel group design, the hospital research pharmacy used block randomization to allocate the patients into either HD, LD, or PL groups. The clinical staff, research personnel, and patients were unaware of the drug allocated to a particular patient (double blinded). The assigned groups of the patients were disclosed by the pharmacy after all the data were acquired, preprocessed, and were ready for group-level statistical analysis.

### Power analysis

In our pilot study [18], we were able to collect useable data from 5 of the 11 patients that were enrolled (45%). In the 5 patients with usable results, the signal of interest had a mean value of −3.764 × 10^−7^ Moles with a standard deviation of 2.135 × 10^−7^ Moles. This resulted in a standardized effect size of 1.763, when using a zero mean for the null case. In order to achieve a 90% power level for this measure, the number of usable data sets is therefore 8 for each group (total of 24 patients). Considering our previous success rate of 45%, approximately 18 patients will need to be enrolled into each group of the study for a total enrollment of 54 participants. However, only 41 patients were able to complete the study, as recruitment was discontinued in March 2020 (a year earlier than projected) due to the coronavirus pandemic in the US. Nonetheless, as part of the original Data and Safety Monitoring Plan, we reviewed the data (blinded) after the first 5 participants and then after every 10 participants to ensure that good quality data were obtained. A Data Safety and Quality Report was submitted to the IRB every time an interim analysis was performed. Each report (total = 5) comprised an evaluation of data quality and a blinded preliminary analysis of the cortical response to cardiac ablation for each patient. This way, when the data collection had to be discontinued in March 2020, we could confirm that adequate data were available to proceed with the unblinding process. The decision to unblind was ultimately made by the principal investigators (BK and DB).

### fNIRS acquisition

**Fig 2:**
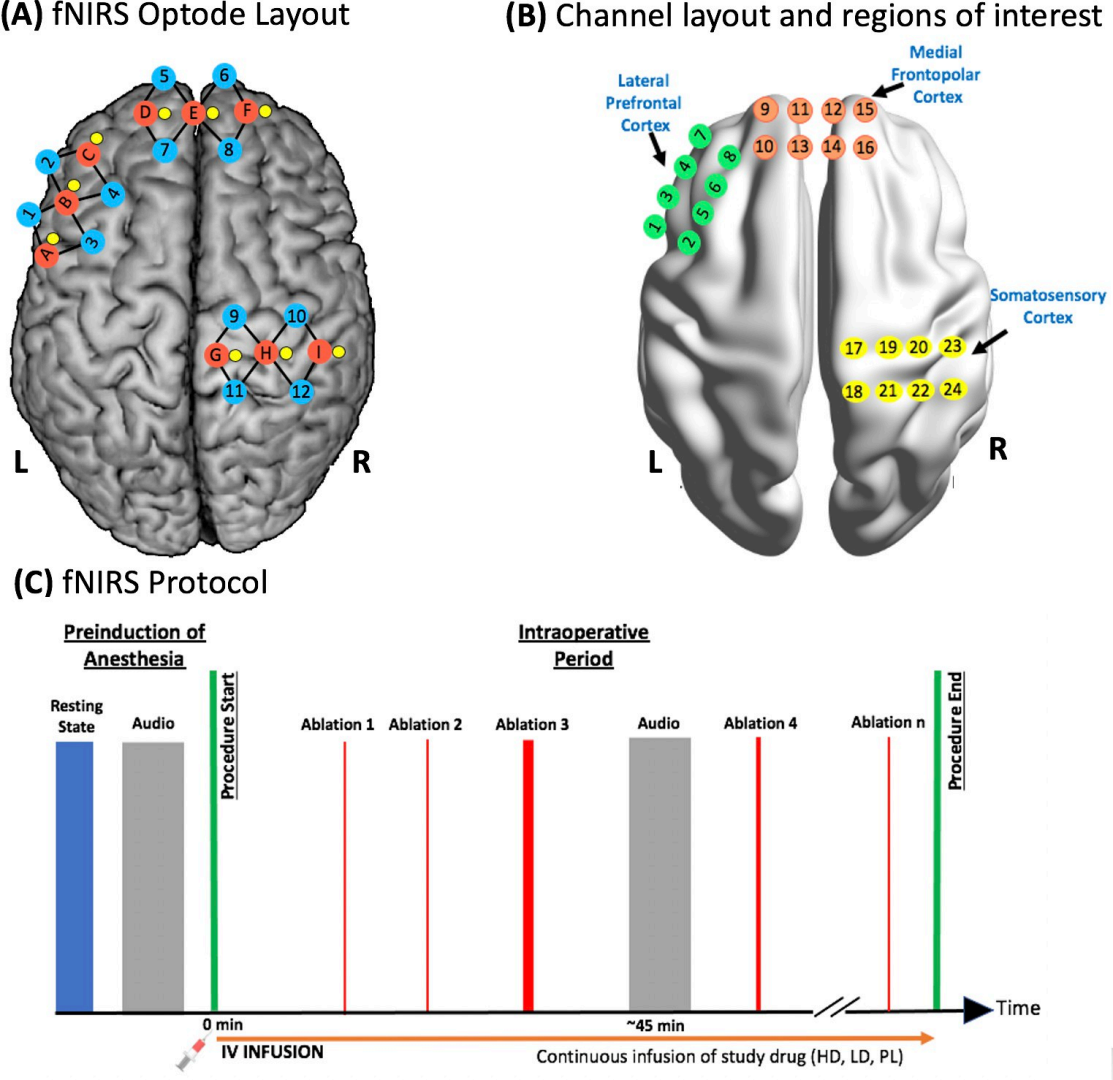
fNIRS optode layout and study paradigm. **(A)** Brain map of customized optode placement where red represent optical sources (sources A to I), blue represent long-separation detectors (detectors 1 to 12, placed 3 cm from the source), and yellow represent the short-separation detectors (0.8 cm from the source). A long-separation detector and source pair form a channel and are represented by the black lines connecting the sources (in red) and detectors (in blue). A short-separation detector (in yellow) and source (in red) pair are called the short-separation channel and measure the extracerebral hemodynamic changes. **(B)** Brain map of 24 channel locations (green, orange, and yellow) and the cortical ROI. **(C)** A resting state and an audio run, each lasting for 200 seconds, were collected before the induction of anesthesia. IV infusion of the test drug (either PL, LD remifentanil, or HD remifentanil) was started at the same time as intraoperative fNIRS data collection. The time, number, and duration of ablations varied between patients and was determined by the cardiologist. Time stamps of each ablation was noted in the fNIRS data. An audio run was collected at least once starting 45 minutes after the initiation of the infusion and fNIRS data collection. fNIRS, functional near-infrared spectroscopy; HD, high-dose; IV, intravenous; LD, low-dose; ROI, region of interest.

Changes in hemoglobin concentration during procedure were recorded using a multichannel continuous wave fNIRS system (CW7, Tech En, Massachusetts, USA) at 690 and 830 nm wavelengths and a sampling frequency of 25 Hz. A customized head probe consisting of 9 optical sources, 12 long-separation optical detectors placed at a distance of 3 cm from the source, and 9 short-separation optical detectors placed at a distance of 0.8 cm from the source [28] was used (described in **Fig 2A**). Of the total 33 channels (a channel being a source and detector pair), 24 channels (indicated by the black lines in **Fig 2A** and numbers in **Fig 2B**) recorded cortical hemoglobin concentration changes, and 9 channels recorded physiological hemoglobin concentration changes from extracerebral tissue (short-separation detectors(yellow) and source(red) pairs in **Fig 2A** form short-separation channels).

### Cortical regions of interest

The 24 channels recorded activity from 3 different cortical regions viz. left lateral prefrontal cortex (left lPFC), mFPC, and right S1 (**Fig 2B**). Moreover, 12 of these 24 channels were subdivided into 6 regions of interest (ROIs) based on their consistent activation/deactivation to acute nociception in previous studies [15,17,18,20]. ROIs were defined by taking the average of 2 channels each: inferior lateral prefrontal cortex (Inf. lPFC, channels 3 to 4), superior lateral prefrontal cortex (Sup. lPFC, channels 5 to 6), inferior medial frontopolar cortex (Inf. mFPC, channels 11 to 12), superior medial frontopolar cortex (Sup. mFPC, channels 13 to 14), anterior superior S1 (Ant. SS1, channels 19 to 20), and posterior superior S1 (Pos. SS1, channels 21 to 22). The ROIs are shown in **Fig 2B** together with the 3 different cortical regions measured.

### Anesthetic and fNIRS protocol

The anesthetic technique was standardized for all patients and the hospital research pharmacy used block randomization to allocate the patients into 3 groups: Group 1: HD remifentanil– 0.5 mcg/kg/min; Group 2: LD remifentanil– 0.25 mcg/kg/min; and Group 3: PL– 0.9% NaCL. The syringes were prepared by the hospital pharmacy, and this randomized controlled trial was double blinded. Following premedication with 2 mg IV midazolam, anesthesia was induced with fentanyl (1.5 mcg/kg up to a maximum of 3 mcg/kg) and a standard dose of propofol titrated to effect. Rocuronium was used for neuromuscular blockade for endotracheal intubation and during the procedure. No additional fentanyl was administered after induction of anesthesia. Anesthesia was maintained with sevoflurane (end-tidal concentration 1% to 4%), adjusting the end-tidal concentration to maintain a bispectral index (BIS) (Medtronic, Minneapolis, Minnesota, USA) value between 40 and 60. The test drug infusion and fNIRS monitoring were started after induction of anesthesia while the patient was being prepped and continued until just after the last ablation.

Data quality of optode placements and fNIRS was evaluated before the induction of anesthesia. A resting-state fNIRS scan was performed for a duration of 200 seconds with an awake but calm patient lying still in the supine position. This was followed by a 200-second fNIRS motor imagination scan, whereby an audio recording was presented to the patient instructing them to begin (Start) imagining squeezing a tennis ball in their left hand and end (Stop) after 15 seconds. The study paradigm is shown in **Fig 2C**. A qualified member of the research team continuously monitored the fNIRS data quality throughout the procedure and time-stamped each ablation attempt. A research nurse and/or research assistant manually documented the time, duration, and mode (radiofrequency (RF) and/or cryoablation) of each ablation attempt. An audio stimulus prompting a motor task (described earlier) was presented as a control to the patient at least 45 minutes after the start of the procedure. The average duration of an electrophysiologic study with catheter ablation of arrhythmias is 3 to 4 hours.

### fNIRS preprocessing

The fNIRS data were preprocessed and analyzed using in-house scripts in MATLAB R2019b platform. The raw fNIRS data of each participant were first converted from intensity measures to optical density measures. Head motion correction was then performed using a wavelet-based algorithm [29,30]. To remove physiological (heart rate and respiration) and other confounding noise sources, a third-order band-pass filter at 0.01 to 0.15 Hz was applied. Using the modified Beer–Lambert law, optical density measures were converted to oxy-, deoxy-, and total-hemoglobin concentrations using *hmrOD2conc* function in the Homer2 toolbox [31]. A linear temporal regression of the resulting concentration of oxygenated hemoglobin (ΔHbO) time series of each channel was regressed using both the nearest short-separation (physiological channel) signal and the global average of all short-separation signals as the nuisance regressor to remove the effect of extracerebral tissue on cortical activity. The residuals of the ΔHbO time series from temporal regression were then used to perform a third-order polynomial fit to regress nonlinear drifts and linear trends before further analysis.

### fNIRS data analysis

The primary outcome measure was the changes in oxyhemoglobin concentration to ablation in PL versus remifentanil groups. Therefore, the LD and HD remifentanil subgroups were combined and compared to the PL group. For those regions that were statistically different between PL and remifentanil, a post hoc analysis was performed to identify any differences between the 2 doses. Secondary outcome measures included the changes in oxyhemoglobin concentration to actual or intended movement, in response to the auditory instruction, and/or the auditory stimuli. Combined analysis of the remifentanil subgroups and sex-based differences were supplemented to the analysis defined in the protocol on an ad hoc basis.

### Cortical response to ablation

The fNIRS hemodynamic response to an ablation event was computed using the block-averaging technique whereby the preprocessed ΔHbO time series was averaged across the total number of ablations for each participant. Since the duration of ablation varied between events and between individuals, each block or trial was defined as the 5 seconds before the start of an ablation event and the 20 seconds following the start of ablation for consistency. Each block was then normalized to the 5 seconds of baseline prior to the start of the ablation in a given block. Hemodynamic-based measures quantified from the block-averaged hemodynamic response to ablation stimuli included (1) Peak ΔHbO (Peak_HbO_), which was defined as the maximum ΔHbO concentration change from 4 seconds to 15 seconds following stimulus after subtracting the average HbO concentration change during the initial 0–3 seconds of stimulus; Peak_HbO_ for deactivation to stimulus was computed on the absolute hemodynamic response; (2) minimum ΔHbO (Nadir_HbO_) was defined as the greatest decrease in ΔHbO concentration in the 15 seconds following the start of ablation; (3) area under the ΔHbO curve (AUC) was defined as the integral of the ΔHbO curve during the 0- to 15-second period following the start of stimulus. The ΔHbO curve was first scaled using the Nadir_HbO_ concentration change for that duration, i.e., the Nadir_HbO_ becomes the 0 baseline for calculation of AUC. Two sample *t* tests were performed to compare the activation measures between PL and drug groups. A statistical threshold of *p* < 0.05, with multiple comparison correction using Benjamini–Hochberg false discovery rate (FDR) approach at an alpha of 0.05 was employed to minimize type I errors [32]. Multiple comparison correction using FDR was applied for comparisons from all 3 measures (Peak_HbO_, Nadir_HbO_, and AUC) together. Results that survived the FDR-p threshold are reported to be significant at FDR-corrected *p* < 0.05. Results with *p*-values > FDR-p threshold did not survive multiple comparison correction. The FDR-p threshold and the original *p*-values are both provided for all comparisons. The 95% confidence intervals (CIs) were generated using false coverage-statement rate that defines the CI coverage corresponding to the FDR-adjusted *p*-values [33]. A post hoc analysis using 2-sample *t* test was performed to identify dose-dependent differences between the remifentanil groups (LD versus HD).

### Sex-related differences in pain response

Hemodynamic measures of activation (Peak_HbO_, Nadir_HbO_, and AUC) during ablation was compared between male (*n* = 16) and female (*n* = 16) participants using a 2-way analysis of variance (ANOVA) with sex (males and females), and drug (drug and PL) as factors. Effect of biological sex was evaluated due to the altered pain sensitivity and treatment outcomes typically found in male versus female patients [34]. Significant effects of sex were obtained using a statistical threshold of *p* < 0.05, and a multiple comparison correction using Benjamini–Hochberg FDR was applied at alpha of 0.05 to account for type I errors [32].The 95% CIs were also adjusted for effects that were significant at FDR-corrected *p* < 0.05 using method proposed by Benjamini and Yekutieli [33].

### Cortical response to nonpainful stimuli

The fNIRS hemodynamic response to auditory stimuli instructing individuals to perform a motor imagery task was also computed using the block averaging technique. The task paradigm lasted for a total of 5 minutes and was presented at least once during the procedure in every participant. A single run with 5 blocks of stimuli was used to calculate hemodynamic response to auditory stimuli during the procedure. A block was defined as the 5 seconds before auditory cue instructing the patient to start the task (lasting a duration of 1 second) and the 29 seconds following the first auditory cue, including the auditory cue to end task at 15 seconds. Hemodynamic-based measures quantified from the block-averaged hemodynamic response were also defined using the peak change in ΔHbO concentration (Peak_HbO_), Nadir of ΔHbO concentration (Nadir_HbO_), and AUC measures described earlier. A mixed ANOVA was performed to compare the activation measures during the 2 types of stimulus between PL and drug groups and their interaction, where “task” is the within-subject factor with 2 levels (audio and pain/ablation) and “group” is the between-subject factor with 2 levels (remifentanil and PL). A statistical threshold of *p* < 0.05 with multiple comparison correction using Benjamini–Hochberg FDR approach at an alpha of 0.05 was once again employed [32]. Multiple comparison correction using FDR was applied for comparisons from each measure (Peak_HbO_, Nadir_HbO_, and AUC) separately. As noted earlier, the 95% CIs were adjusted for effects that were significant at FDR-corrected *p* < 0.05 using method proposed by Benjamini and Yekutieli [33].

## Results

### Cortical response to nonpainful stimuli

The demographic and procedural characteristics of the 41 participants recruited and scanned from October 2016 to March 2020 are summarized in **Table 1**. Catheter-based ablations were performed in all 41 participants; however, 7 participants were excluded due to poor fNIRS signal quality, (i.e., no visible heart rate in signal indicating poor scalp-optode contact) and 2 participants were excluded because they received only cryoablations (to reduce heterogeneity in surgical procedure as cryoablation is reported to be less painful [35–38]). This step was performed before the data set was unblinded. The mean ± SD age of the remaining participants was 15.8 ± 2.1 (*n* = 32), corresponding to 16.0 ± 25 years for the HD group (*n* = 11), 15.7 ± 1.8 years for the LD group (*n* = 12), and 15.5 ± 2.4 years for the PL group (*n* = 9). Baseline patient characteristics were evaluated using standardized mean difference (SMD) to identify any group imbalance that could confound the intervention effect. The SMD of age was 0.164 (or 16.4%), suggesting an adequate balance in the age of the 2 groups. We also found no significant differences in age between the 3 subgroups using 1-way ANOVA (F (2,29) = 0.15, *p* = 0.86). There are 11 males (47.8%) and 12 females (52.2%) in the remifentanil group and 5 males (55.6%) and 4 females (44.4%) in the PL group. The SMD of the proportion of male and female participants in the 2 groups is 0.15 (or 15%), indicating a good balance between the 2 groups. Although a small imbalance (SMD = 0.291 or 29.1%) in the number of ablations was found between the 2 groups, both 2-sample *t* tests (*p* = 0.46) and 1-way ANOVA (F (2,29) = 0.29, *p* = 0.74) showed no differences in the number of ablations between drug versus PL groups, and HD, LD, and PL subgroups respectively. The average number of ablations ± SD in each group was 10.4 ± 9.3 in the HD group, 9.5 ± 10.8 in the LD group, and 7.4 ± 4.3 in the PL group. The mean (±SD) time from the administration of fentanyl to the first ablation was 120 ± 33 minutes for the remifentanil group (*n* = 21; note, time of fentanyl administration is unavailable for patient 023 and 031), while the mean (±SD) time from the administration of fentanyl to the first ablation was 137 ± 56 minutes in the PL group (*n* = 9).

**Table 1:** Demographic and procedural data (*n* = 41).*

Patient	Age (years)	Sex	Body weight (kgs)	Ablation type	Number of RF ablations	Total duration of RF ablation (s)	Number of CRYOs	Vascular access and number of catheters
**HD group**								
1	16	M	76.1	RF + CRYO	30	950.24	3	RFV × 1, LFV × 3
4	20	F	49	RF	24	641.28	0	RFV × 1, LFV × 3
8	21	F	84.8	RF	7	215.28	0	RFV × 1, LFV × 3
11	14	M	57.5	RF	2	117.5	0	RFV × 1, LFV × 3
15	16	F	76.9	RF	3	90	0	RFV × 2, LFV × 2
17	14	M	80.9	RF	6	303.28	0	RFV × 1, LFV × 3
20	15	M	58.5	RF	3	182	0	RFV × 2, LFV × 2
23	16	F	67.6	RF	10	388	0	RFV × 1, LFV × 3
26	16	F	65.9	RF	14	204	0	RFV × 2, LFV × 3, RIJV × 1
27	15	M	78.3	RF	1	30	0	RFV × 1, LFV × 3, RFA × 1
33	15	M	45.4	RF	6	126	0	RFV × 1, LFV × 3, RFA × 1
34	18	M	81.1	RF	17	532	0	RFV × 2, LFV × 3
37	13	F	74.4	RF	9	318	0	RFV × 1, LFV × 3
**LD group**								
3	17	M	64.5	RF + CRYO	11	320.16	12	RFV × 1, LFV × 3
5	18	F	48.4	RF + CRYO	2	23	6	RFV × 1, LFV × 3, RIJV × 1, RFA × 1
9	18	F	75.1	RF	7	323	0	RFV × 1, LFV × 3
10	13	F	53.4	RF	2	90	0	RFV × 1, LFV × 3
14	17	M	55.5	RF	10	395.76	0	RFV × 3, RIJV × 1
18	14	F	60.6	RF	1	61	0	RFV × 1, LFV × 3
19	17	F	70.1	RF	4	184	0	RFV × 1, LFV × 3
22	13	M	47.3	RF	1	60	0	RFV × 1, LFV × 2
25	16	M	48.4	RF	26	407	0	RFV × 1, LFV × 3
29	17	F	62.4	CRYO	0	0	4	RFV × 1, LFV × 3
31	16	F	74.9	RF	26	623	0	RFV × 2, LFV × 3, RIJV × 1
38	16	F	64	RF	29	889	0	RFV × 1, LFV × 3
39	14	F	62.5	RF	4	169	0	RFV × 1, LFV × 3
40	17	M	74.7	RF	3	120	0	RFV × 1, LFV × 3, RIJV × 1
**PL group**								
2	12	M	93.6	RF	6	192	0	RFV × 1, LFV × 3
6	17	F	49.3	RF	3	20.6	0	RFV × 1, LFV × 3, RFA × 1
7	14	F	38.2	RF	7	176.12	0	RFV × 2, LFV × 2, RIJV × 1
12	16	M	58.7	RF	16	318	0	RFV × 2, LFV × 2
13	18	M	44.8	RF + CRYO	14	652.4	2	RFV × 1, LFV × 3
16	17	M	81.2	RF	2	72	0	RFV × 1, LFV × 3, RIJV × 1
21	12	M	58.9	RF	5	175	0	RFV × 1, LFV × 3
24	16	F	70	RF	12	239	0	RFV × 1, LFV × 3
28	16	M	61.4	RF	6	260	0	RFV × 1, LFV × 3
30	12	M	58.6	CRYO	0	0	1	RFV × 2, LFV × 2, RIJV × 1
32	12	M	56.6	RF	4	185	0	RFV × 1, LFV × 3, RIJV × 1
35	19	F	56.6	RF	8	313	0	RFV × 2, LFV × 3
36	19	F	87.9	RF	5	197	0	RFV × 2, LFV × 3
41	17	M	56.1	RF	8	358	0	RFV × 1, LFV × 3, RIJV ×1

*Participants 3, 13, 15, 26, 28, 32, and 36 were excluded from data analysis due to poor fNIRS signal quality, and participants 29 and 30 were excluded because they did not receive RF ablation. The decision to exclude data was made before unblinding the groups.

CRYO, cryoablation; fNIRS, functional near-infrared spectroscopy; HD, high-dose; LD, low-dose; LFV, left femoral vein; PL, placebo; RF, radiofrequency; RFV, right femoral vein; RIJV, right internal jugular vein.

There were no serious adverse events. As is typical for electrophysiologic procedures, heart rate varied widely during arrhythmia mapping and administration of isoproterenol. Phenylephrine or ephedrine was deemed necessary to support hemodynamics in 35.7%, 50%, and 76.9% of the PL, LD, and HD groups, respectively. In the recovery room, 4 patients (9.8%; 2 in LD group and 1 each in PL and HD groups) had both nausea and vomiting, while 4 patients (9.8%; 3 in PL group and 1 in LD group) had nausea only.

### Cortical response to painful events (ablations)

**Fig 3:**
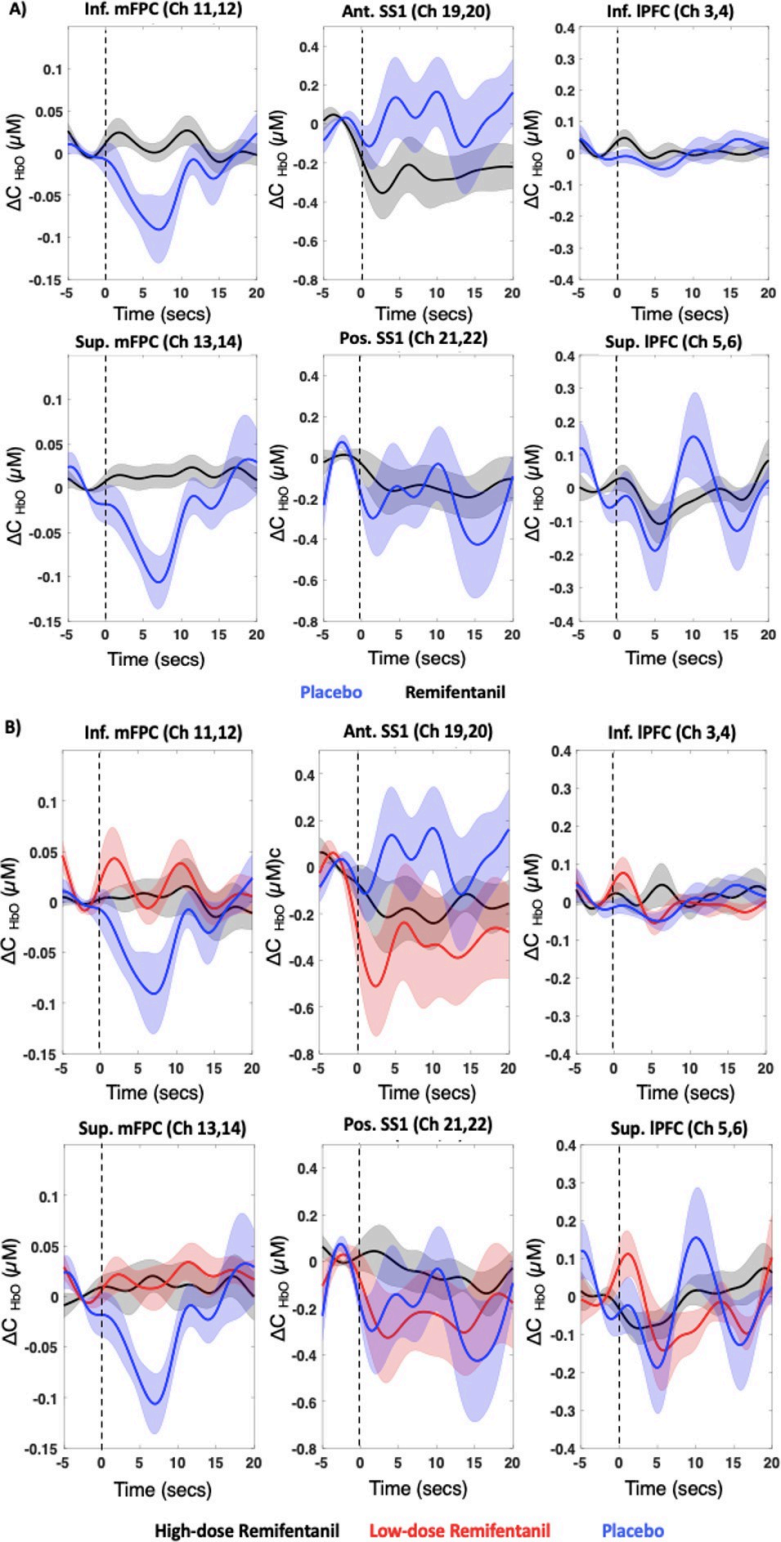
Group-averaged hemodynamic response to ablation in the 6 ROIs. **(A)** Remifentanil (HD + LD = black) and PL (blue) groups. **(B)** Remifentanil subgroups (HD = black; LD = red) and PL (blue) group. The dotted black line indicates the start of ablation. The shaded region represents the standard error of mean. Ant. SS1, anterior superior somatosensory cortex; HD, high-dose; Inf. lPFC, inferior lateral prefrontal cortex; Inf. mFPC, inferior medial frontopolar cortex; LD, low-dose; PL, placebo; Pos. SS1, posterior superior somatosensory cortex; ROI, region of interest; Sup. lPFC, superior lateral prefrontal cortex; Sup. mPFC, superior medial frontopolar cortex.

The group-averaged hemodynamic response to ablations for the 6 ROIs is presented in **Fig 3**, and the group-averaged hemodynamic response to only the first ablation event is presented in **S1 Fig** of the Supporting information. The inferior and superior regions of the medial FPC (Brodmann Area 10) in the PL group exhibited a deactivation (decrease in ΔHbO) in the 10 seconds following ablation in contrast to a net positive or no change in the patients receiving remifentanil (**Fig 3A**) and between the HD and LD groups (**Fig 3B**). With respect to the Ant. SS1, the PL group had the expected activation response with an increase in ΔHbO as opposed to the large decrease and net negative change seen with remifentanil groups (see **Fig 3A and 3B**). Interestingly, the HD group showed the least variation in ΔHbO in response to ablation in the somatosensory ROIs. A similar differential response to the ablation event between the PL and remifentanil groups was observed in the superior lateral PFC.

**Fig 4:**
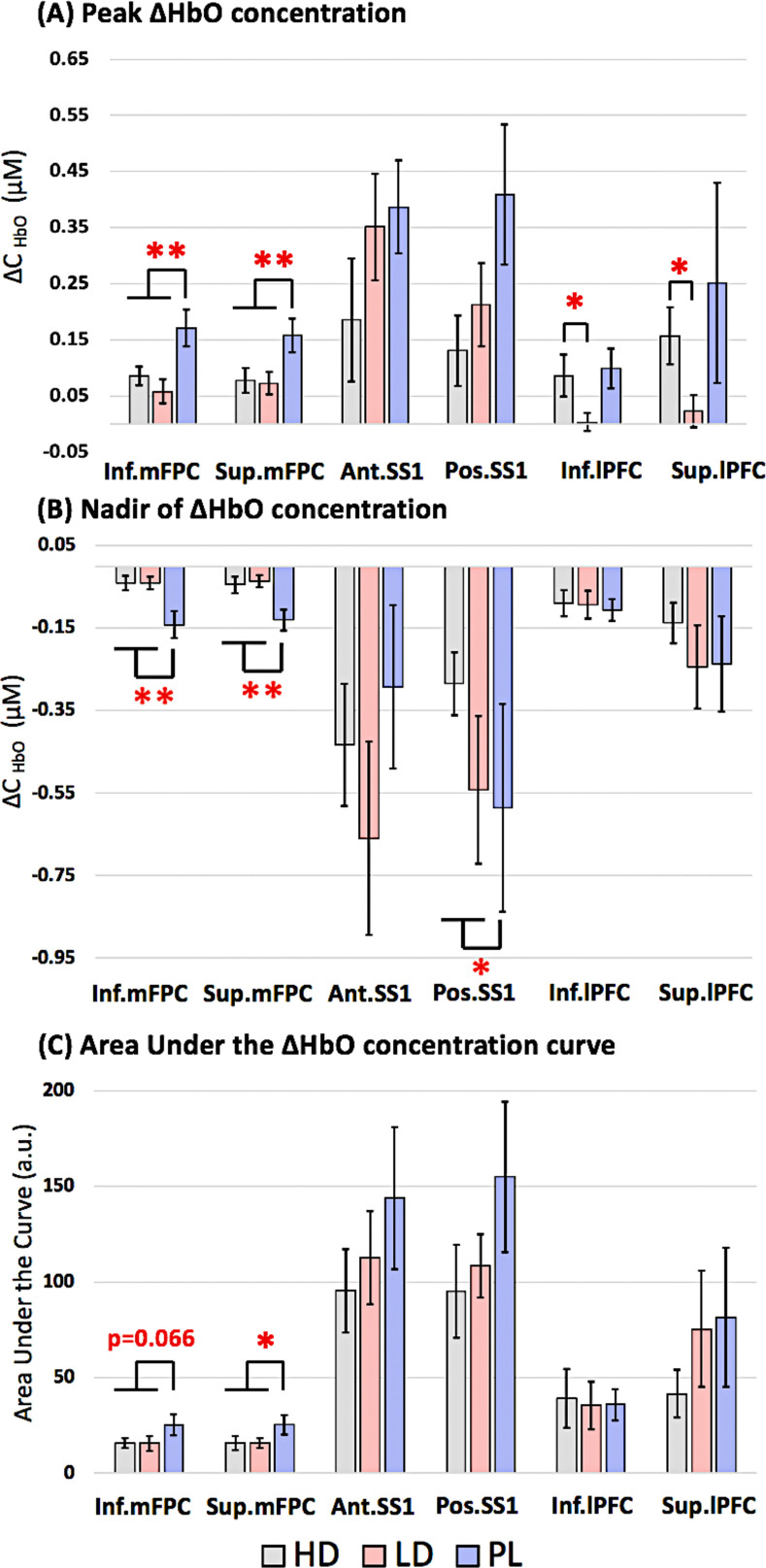
Group-level hemodynamic measures of activation to ablation in PL and 2 subgroups of remifentanil. **(A)** Peak_HbO_ concentration. **(B)** Nadir of ΔHbO concentration. **(C)** AUC. The plot on the right describes the measures calculated from a standard hemodynamic response. ** indicates significant effects between PL and remifentanil using 2-sample *t* tests at FDR-corrected *p* < 0.05. The FDR-p threshold = 0.008. * indicates significant effects using 2-sample *t* tests at uncorrected *p* < 0.05. Error bars represent the standard error of mean. Ant. SS1, anterior superior somatosensory cortex; AUC, area under the ΔHbO curve; FDR, false discovery rate; Inf. lPFC, inferior lateral prefrontal cortex; Inf. mFPC, inferior medial frontopolar cortex; Peak_HbO_, Peak ΔHbO; PL, placebo; Pos. SS1, posterior superior somatosensory cortex; Sup. lPFC, superior lateral prefrontal cortex; Sup. mPFC, superior medial frontopolar cortex.

The mean cortical measures of activation (Peak_HbO_, Nadir_HbO_, and AUC) were similar between the LD and HD groups in the mFPC regions, but appeared to exhibit a dose-based effect in the somatosensory and superior lPFC regions where PL, followed by LD, and then HD remifentanil groups presented with the greatest to least activation to ablation (**Fig 4**). The Peak_HbO_ measure shown in **Fig 4A** of inferior mFPC (mean difference [MD] (REM-PL) = −1.138, adj. 95% CI = −2.062, −0.214, 95% CI 0.441, 1.835, *p* = 0.002, Cohen’s d = 1.193 and superior mFPC (MD (REM-PL) = −0.999, adj. 95% CI = −1.961, −0.036, *p* = 0.008, Cohen’s d = 1.031) was greater in the PL group than the remifentanil group at FDR-corrected *p* < 0.05 (FDR-p threshold = 0.008). Posterior SS1 (*p* = 0.03) of the PL group exhibited a similar response, where the Peak_HbO_ measure to ablation was greater than either of the remifentanil groups. Similarly, the Nadir_HbO_ measure of inferior mFPC (MD (REM-PL) = 1.229, adj. 95% CI = 0.334, 2.124, *p* < 0.001, Cohen’s d = 1.26) and superior mFPC (MD (REM-PL) = 1.206, adj. 95% CI = 0.303, 2.109, *p* = 0.001, Cohen’s d = 1.26) was greater (i.e., greater decrease in HbO) in PL group when compared to the remifentanil group at FDR-corrected *p* < 0.05 (**Fig 4B**). The FDR-p threshold was 0.001. The AUC, although not statistically significant after multiple comparison correction, displayed a similar effect in the inferior mFPC (*p* = 0.06) and superior mFPC (*p* = 0.04) regions (**Fig 4C**). Post hoc analysis using *t* tests showed a greater Peak_HbO_ to ablation in the inferior lPFC (*p* = 0.04) and superior lPFC (*p* = 0.02) of the HD group when compared to the LD group at uncorrected *p* < 0.05. No other notable differences were found between groups in other ROIs or between drug subgroups (LD versus HD). The average Peak_HbO_, Nadir_HbO_, and AUC measures during ablation in each of the groups and independent sample *t* test outputs are provided in **Table 2**.

**Table 2:** Results of independent sample *t* test comparing cortical measures of activation between remifentanil versus PL groups.

		Peak_HbO_		Nadir of ΔHbO		AUC	
Region		Mean ± SEM	*p*-Value	Mean ± SEM	*p*-Value	Mean ± SEM	*p*-Value
**Inf.mFPC**	**HDR**	0.085 ± 0.016	*p* = 0.002** Adj. 95% CI = (−2.062, −0.214) Cohen’s d = 1.193	−0.040 ± 0.017	*p* < 0.001** Adj. 95% CI = (0.334, 2.124) Cohen’s d = 1.260	15.683 ± 2.425	0.06
	**LDR**	0.057 ± 0.021		−0.041 ± 0.015		15.706 ± 3.909	
	**PL**	0.170 ± 0.032		−0.141 ± 0.033		25.196 ± 5.495	
**Sup.mFPC**	**HDR**	0.077 ± 0.021	*p* = 0.008** Adj. 95% CI = (−1.961, −0.036) Cohen’s d = 1.031	−0.044 ± 0.020	*p* = 0.001** Adj. 95% CI = (0.303, 2.109) Cohen’s d = 1.260	15.634 ± 3.623	0.04
	**LDR**	0.072 ± 0.019		−0.036 ± 0.015		15.731 ± 2.489	
	**HDR**	0.157 ± 0.030		−0.130 ± 0.025		25.399 ± 5.024	
**Ant.SS1**	**HDR**	0.185 ± 0.109	0.37	−0.433 ± 0.147	0.32	95.376 ± 21.719	0.26
	**LDR**	0.350 ± 0.095		−0.659 ± 0.234		112.650 ± 24.229	
	**PL**	0.386 ± 0.082		−0.292 ± 0.198		143.725 ± 37.211	
**Pos. SS1**	**HDR**	0.130 ± 0.062	0.03	−0.284± 0.076	0.46	95.199 ± 24.330	0.12
	**LDR**	0.212 ± 0.074		−0.541 ± 0.179		108.374 ± 16.526	
	**PL**	0.408 ± 0.124		−0.585± 0.252		155.067 ± 39.318	
**Inf.lPFC**	**HDR**	0.086 ± 0.037	0.17	−0.089 ± 0.031	0.72	39.077 ± 15.493	0.93
	**LDR**	0.003 ± 0.016		−0.093 ± 0.033		35.496 ± 12.386	
	**PL**	0.099 ± 0.035		−0.105 ± 0.026		35.787 ± 8.049	
**Sup.lPFC**	**HDR**	0.156 ± 0.050	0.180	−0.137 ± 0.048	0.70	41.614 ± 12.418	0.53
	**LDR**	0.022 ± 0.028		−0.243 ± 0.100		75.484 ± 30.408	
	**PL**	0.250 ± 0.178		−0.237 ± 0.115		81.577 ± 36.490	

** indicates regions that were significant after multiple comparison correction at FDR-corrected *p* < 0.05. The FDR-p threshold was 0.008. The adj. 95% CI and Cohen’s d effect size for these regions are provided. Adj. 95% CIs represent the false coverage-statement rate adjusted CIs that correspond to the FDR-adjusted *p*-values.

Adj. 95% CI, adjusted 95% CI; Ant. SS1, anterior superior somatosensory cortex; AUC, area under the ΔHbO curve; FDR, false discovery rate; HDR, high-dose remifentanil; Inf. lPFC, inferior lateral prefrontal cortex; Inf. mFPC, inferior medial frontopolar cortex; LDR, low-dose remifentanil; Peak_HbO_, Peak ΔHbO; PL, placebo; Pos. SS1, posterior superior somatosensory cortex; SEM, standard error of mean; Sup. lPFC, superior lateral prefrontal cortex; Sup. mPFC, superior medial frontopolar cortex.

### Sex-related differences in cortical response to painful events (ablations)

**Fig 5:**
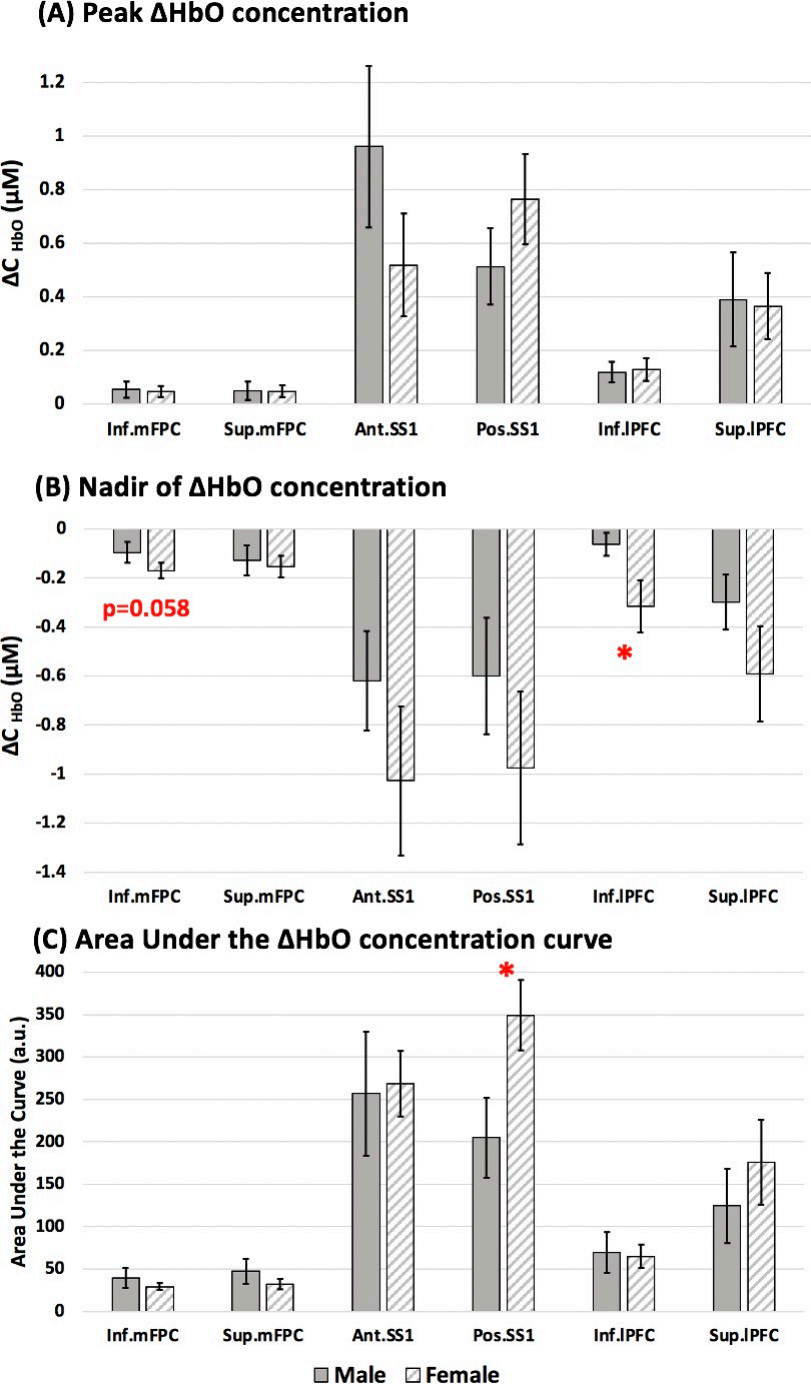
Sex-related effects in response to ablation in male versus female patients under general anesthesia. **(A)** Peak_HbO_ concentration. **(B)** Nadir of ΔHbO concentration. **(C)** AUC. * indicates significant effects between male and female patients using 2-sample *t* tests at uncorrected *p* < 0.05. Error bars represent the standard error of mean. Ant. SS1, anterior superior somatosensory cortex; AUC, area under the ΔHbO curve; Inf. lPFC, inferior lateral prefrontal cortex; Inf. mFPC, inferior medial frontopolar cortex; Peak_HbO_, Peak ΔHbO; Pos. SS1, posterior superior somatosensory cortex; Sup. lPFC, superior lateral prefrontal cortex; Sup. mPFC, superior medial frontopolar cortex.

No significant differences in age (*p* > 0.05) or ratio of PL to remifentanil (*X*^2^ (2, *N* = 32) = 0.1546, *p* = 0.69) were found between males and females. An ANOVA was performed comparing the cortical activation measures to ablation between male and female participants while accounting for the drug or PL administered. A greater Peak_HbO_ measure to ablation was observed in the posterior SS1 (*p* = 0.01) of female patients when compared to males at an uncorrected *p* < 0.05 threshold (see **Fig 5A**). A similar effect was also observed for other measures (Nadir_HbO_ and AUC in **Fig 5B and 5C**). The mean activation measures of all the regions are summarized in **S1 Table**. No significant differences were noted after multiple comparison correction. Assuming hormonal profiles may play a role in pain response, we excluded individuals younger than 14 years of age (n_males_ = 14, n_females_ = 14); this did not affect the differences observed between male and female groups before exclusion (shown in **S3 Fig**).

### Cortical response to nonpainful procedure under anesthesia (auditory stimuli)

**Fig 6:**
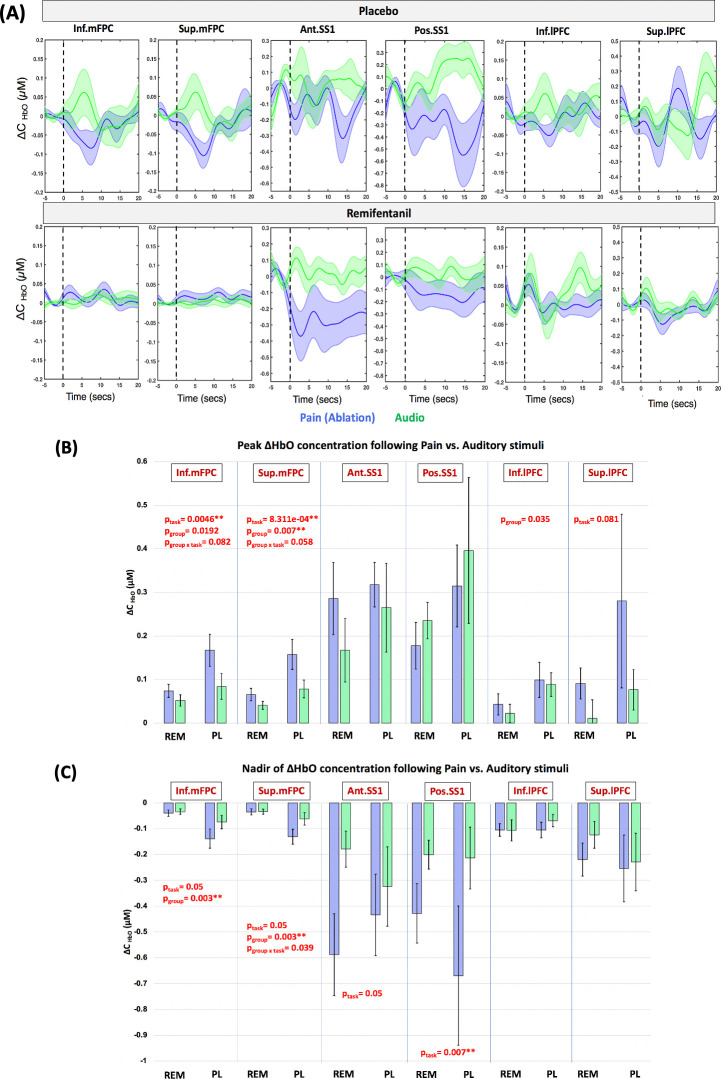
Hemodynamic response to painful versus auditory stimuli under general anesthesia. **(A)** Block-averaged hemodynamic response to pain/ablation (blue) and auditory (green) stimuli in the 2 groups for the 6 regions of the interest. Shaded areas represent the standard error of mean. **(B)** Peak_HbO_ concentration when compared to baseline, following painful versus auditory stimuli in the 6 ROIs classified based on drug group. Mixed ANOVA revealed significant effects at FDR-corrected *p* < 0.05 (FDR-p threshold = 0.007). **(C)** Nadir of ΔHbO concentration following painful versus auditory stimuli in the 6 ROIs classified based on drug group. Mixed ANOVA revealed significant effects at FDR-corrected *p* < 0.05 (FDR-p threshold = 0.007). p_task_ indicates the *p*-value for mean effect of task (pain versus audio); p_group_ indicates the *p*-value for mean effect of drug group (PL versus remifentanil); and p_group × task_ indicates the *p*-value for interaction effect of task and drug group (Pain versus Audio in PL and remifentanil). ** indicates significant effects at FDR-corrected *p* < 0.05. Error bars indicate the standard error of mean. ANOVA, analysis of variance; Ant. SS1, anterior superior somatosensory cortex; FDR, false discovery rate; Inf. lPFC, inferior lateral prefrontal cortex; Inf. mFPC, inferior medial frontopolar cortex; Peak_HbO_, Peak ΔHbO; Pos. SS1, posterior superior somatosensory cortex; ROI, region of interest; Sup. lPFC, superior lateral prefrontal cortex; Sup. mPFC, superior medial frontopolar cortex.

Only 28 patients (88%; 20 with remifentanil, 8 with PL) received the auditory paradigm during the procedure due to technical difficulties (software error, unable to setup auditory paradigm due to time restriction, etc.). The group-averaged hemodynamic response from −5 seconds to 20 seconds following the auditory and ablation events stratified by group are presented in **Fig 6A**. Auditory stimuli in the PL group elicited a net positive increase in ΔHbO of the inferior and superior mFPC channels in contrast to ablation that produced a net decrease, as shown by the green and blue curves in the top panel of **Fig 6A**. Auditory stimuli were also associated with a double peak in HbO response in several ROIs (see lPFC and SS1 of PL and remifentanil groups shown in **Fig 6A**), expected in response to Start and Stop auditory cue at 0 and 15 seconds.

A mixed ANOVA was performed to test the effect of stimulus type (ablation versus auditory), group (remifentanil versus PL), and their interaction on the Peak_HbO_, Nadir_HbO_, and AUC measures of HbO change following stimulation. The FDR-p threshold for both PeakHbO and NadirHbO was 0.007. There were no effects of the stimulus, group, or their interaction on the AUC measures significant after multiple comparison corrections.

### Effect of stimulus type

In general, ablation (painful stimuli) resulted in greater HbO measures of activation than the auditory stimuli in the cortical regions studied. The Peak_HbO_ of the inferior mFPC (MD (P-A) = 0.053, adj. 95% CI = 0.004, 0.101, *p* = 0.004, partial eta-squared (η_p_^2^ = 0.27) and superior mFPC (MD (P-A) = 0.052, adj. 95% CI = 0.013, 0.091, *p* < 0.001, η_p_^2^ = 0.354) regions were greater in response to ablation than auditory stimulus in both groups at FDR-corrected *p* < 0.05. No other regions survived multiple comparison correction. The results of the test are summarized in **Table 3** and **Fig 6B**. The AUC measures of anterior SS1 (*p* = 0.02), posterior SS1 (*p* = 0.003), and superior lPFC (*p* = 0.02) were also greater during ablation than auditory stimulation but failed to survive multiple comparison correction (**S4 Fig**). Similarly, Nadir_HbO_ of Pos. SS1 (MD (P-A) = −0.342, adj. 95% CI = −0.680, −0.004, *p* = 0.007, η_p_^2^ = 0.243) exhibited greater decrease to ablation when compared to auditory stimulus at FDR-corrected *p* < 0.05. Nadir_HbO_ of inferior mFPC (*p* = 0.05) and superior mFPC (*p* = 0.03) also differed between the 2 stimulus types when evaluated without multiple comparison correction. The results of the test comparing Nadir_HbO_ are summarized in **Table 4** and **Fig 6C**.

**Table 3:** Results of mixed ANOVA comparing Peak_HbO_ measure between painful stimulation and auditory stimulation in remifentanil and PL groups.

Region		Mean ± SEM		*p*-Value
		Remifentanil	PL	
**Inf. mFPC**	**Pain**	0.073 ± 0.014	0.167 ± 0.036	p_group_ = 0.01 p_task_ = 0.004** Adj. 95% CI = (0.004, 0.101) η_p_^2^ = 0.270 p_taskxgroup_ = 0.08
	**Audio**	0.051 ± 0.012	0.084 ± 0.029	
**Sup. mFPC**	**Pain**	0.065 ± 0.014	0.157 ± 0.034	p_group_ = 0.007** Adj. 95% CI = (−0.128, −0.002) η_p_^2^ = 0.248 p_task_ < 0.001** Adj. 95% CI = (0.013, 0.091) η_p_^2^ = 0.354 p_taskxgroup_ = 0.05
	**Audio**	0.040 ± 0.009	0.078 ± 0.020	
**Ant. SS1**	**Pain**	0.285 ± 0.082	0.317 ± 0.051	p_group_ = 0.49 p_task_ = 0.38 p_taskxgroup_ = 0.73
	**Audio**	0.167 ± 0.072	0.264 ± 0.101	
**Pos. SS1**	**Pain**	0.177 ± 0.053	0.314 ± 0.093	p_group_ = 0.14 p_task_ = 0.33 p_taskxgroup_ = 0.86
	**Audio**	0.235 ± 0.035	0.395 ± 0.167	
**Inf. lPFC**	**Pain**	0.043 ± 0.024	0.099 ± 0.040	p_group_ = 0.03 p_task_ = 0.61 p_taskxgroup_ = 0.87
	**Audio**	0.022 ± 0.021	0.088 ± 0.027	
**Sup. lPFC**	**Pain**	0.091 ± 0.035	0.280 ± 0.199	p_group_ = 0.11 p_task_ = 0.08 p_taskxgroup_ = 0.43
	**Audio**	0.011 ± 0.042	0.076 ± 0.046	

** indicates effects that were significant after multiple comparison correction at FDR-corrected *p* < 0.05. The FDR-p threshold was 0.007. The adj. 95% CI and partial eta-squared (η_p_^2^) values of these regions are provided. Adj. 95% CIs are the false coverage-statement rate adjusted CIs that correspond to the FDR-corrected *p* < 0.05. “P” represents the painful condition (ablation procedure), and “A” represents the auditory condition. p_task_ is the *p*-value for mean effect of task (pain versus audio); p_group_ is the *p*-value for mean effect of drug group (PL versus remifentanil); and p_group × task_ is the *p*-value for interaction effect of task and drug group (pain versus audio in PL and remifentanil).

Adj. 95% CI, adjusted 95% CI; ANOVA, analysis of variance; Ant. SS1, anterior superior somatosensory cortex; FDR, false discovery rate; Inf. lPFC, inferior lateral prefrontal cortex; Inf. mFPC, inferior medial frontopolar cortex; Peak_HbO_, Peak ΔHbO; PL, placebo; Pos. SS1, posterior superior somatosensory cortex; SEM, standard error of mean; Sup. lPFC, superior lateral prefrontal cortex; Sup. mPFC, superior medial frontopolar cortex.

**Table 4:** Results of mixed ANOVA comparing Nadir of ΔHbO measure between painful stimulation and auditory stimulation in Remifentanil and PL groups.

Region		Mean ± SEM		*p*-Value (adj. 95% CI lower bound, upper bound)
		Remifentanil	PL	
**Inf.mFPC**	P	−0.040 ± 0.012	−0.138 ± 0.037	p_group_ = 0.003** Adj. 95% CI = (0.009, 0.130) η_p_^2^ = 0.291 p_task_ = 0.05 p_taskxgroup_ = 0.10
	A	−0.034 ± 0.011	−0.074 ± 0.026	
**Sup.mFPC**	P	−0.035 ± 0.011	−0.131 ± 0.028	p_group_ = 0.003** Adj. 95% CI = (0.008, 0.116) η_p_^2^ = 0.291 p_task_ = 0.03 p_taskxgroup_ = 0.03
	A	−0.034 ± 0.009	−0.062 ± 0.024	
**Ant.SS1**	P	−0.588 ± 0.158	−0.433 ± 0.157	p_group_ = 0.97 p_task_ = 0.05 p_taskxgroup_ = 0.24
	A	−0.179 ± 0.069	−0.323 ± 0.153	
**Pos. SS1**	P	−0.428 ± 0.115	−0.669 ± 0.269	p_group_ = 0.45 p_task_ = 0.007** Adj. 95% CI = (−0.680, −0.004) η_p_^2^ = 0.243 p_taskxgroup_ = 0.34
	A	−0.200 ± 0.056	−0.213 ± 0.119	
**Inf.lPFC**	P	−0.105 ± 0.024	−0.105 ± 0.030	p_group_ = 0.70 p_task_ = 0.53 p_taskxgroup_ = 0.51
	A	−0.106 ± 0.040	−0.068 ± 0.023	
**Sup.lPFC**	P	−0.220 ± 0.063	−0.254 ± 0.229	p_group_ = 0.49 p_task_ = 0.34 p_taskxgroup_ = 0.57
	A	−0.124 ± 0.051	−0.229 ± 0.111	

** indicates effects that were significant after multiple comparison correction at FDR-corrected *p* < 0.05. The FDR-p threshold was 0.007. The adj. 95% CI and partial eta-squared values (η_p_^2^) of these regions are provided. Adj. 95% CIs are the false coverage-statement rate adjusted CIs that correspond to the FDR-corrected *p* < 0.05. “P” represents the painful condition (ablation procedure), and “A” represents the auditory condition. p_task_ is the *p*-value for mean effect of task (Pain versus Audio); p_group_ is the *p*-value for mean effect of drug group (PL versus remifentanil); and p_group × task_ is the *p*-value for interaction effect of task and drug group (Pain versus Audio in PL and remifentanil).

Adj. 95% CI, adjusted 95% CI; ANOVA, analysis of variance; Ant. SS1, anterior superior somatosensory cortex; FDR, false discovery rate; Inf. lPFC, inferior lateral prefrontal cortex; Inf. mFPC, inferior medial frontopolar cortex; PL, placebo; Pos. SS1, posterior superior somatosensory cortex; SEM, standard error of mean; Sup. lPFC, superior lateral prefrontal cortex; Sup. mPFC, superior medial frontopolar cortex.

### Effect of drug group

PL group when compared to remifentanil group was associated with greater HbO measures of activation to stimulation under anesthesia. Specifically, the Peak_HbO_ of the inferior mFPC (*p* = 0.019) and superior mFPC (MD (REM-PL) = −0.092, adj. 95% CI = −0.680, −0.004, *p* = 0.007, η_p_^2^ = 0.248) regions were greater in PL group when compared to the remifentanil group for both the stimulus types at FDR-corrected *p* < 0.05. The inferior lPFC (*p* = 0.03) region also exhibited greater Peak_HbO_ in PL when compared to remifentanil group, however, failed to survive multiple comparison correction. AUC measures of inferior mFPC (*p* = 0.01) and superior mFPC (*p* = 0.009) were also greater in PL group when compared to the remifentanil group at uncorrected *p* < 0.05 (**S4 Fig**). Likewise, the Nadir_HbO_ of inferior mFPC (MD (REM-PL) = 0.098, adj. 95% CI = 0.009, 0.130, *p* = 0.003, η_p_^2^ = 0.291) and superior mFPC (MD (REM-PL) = 0.096, adj. 95% CI = 0.008, 0.116, *p* = 0.003, η_p_^2^ = 0.291) was greater in PL group irrespective of the stimulus type at FDR-corrected *p* < 0.05.

### Interaction of stimulus type and drug group

An interaction effect of stimulus type and group on Peak_HbO_ and Nadir_HbO_—although not statistically different potentially due to small sample size—was noted in the inferior and superior mFPC channels. The difference between stimulus type (ablation versus auditory) was greater in the PL group when compared to the remifentanil group (see REM and PL in **Fig 6B and 6C**).

## Discussion

### Summary of findings

To the best of our knowledge, this is the first paper to report the analgesic effects of an opioid using fNIRS measures of cortical responses in patients under general anesthesia. During catheter ablation, considered a painful process, responses were observed in the mFPC and S1 consistent with pain as determined by our prior measures of nociceptive evoked responses in awake, sedated, and anesthetized patients. Furthermore, remifentanil resulted in a decrease in the magnitude of these responses consistent with opioid effects similar to that observed in response to morphine in awake participants [17], but remifentanil did not completely abolish the signal. However, the greatest effect of opioid on the fNIRS response to ablation was found for the higher dose (0.5 mcg/kg/min) followed by the lower dose (0.25 mcg/kg/min) of the remifentanil, especially in the S1. Importantly, auditory responses in mFPC were opposite to presumed nociceptive responses (as shown in PL group). As such, the 2 processes suggests that sensory processing is present during general anesthesia.

In this present study, we follow up on a prior report evaluating fNIRS signals during catheter ablation of arrhythmias in which we found a significant decrease in signal in the mFPC in response to RF or cryoablation [18]. The signal change observed in this study mirrored our previous observations relating to fNIRS signals from noxious stimulation in awake healthy volunteers to electrical painful stimuli [15,20] and in patients experiencing pain to colon insufflation under sedation [15]. Furthermore, we previously showed a decrease in the fNIRS pain response following morphine administration [17] and now report on the potential effects of an opioid (remifentanil) on the pain response from RF ablation. Our results demonstrate a number of findings related to the anesthetic process including the following: (1) Nociceptive/pain effects—positive fNIRS response in S1 and lPFC to ablation that are anticorrelated to mFPC in the PL group and similar to those observed previously [18] to painful/nociceptive stimuli in awake, sedated, and anesthetized individuals [15,18,20]; (2) Opioid effects—when compared to PL group, there is a decrease in deactivation of mFPC to ablation in the remifentanil group and a decrease in activation of S1 to ablation in the remifentanil group. Even though not significant using statistical comparisons, the average hemodynamic response showed that the greatest effect of opioid to ablation was found for the higher dose (0.5mcg/kg/min), which shows least change to ablation, followed by the lower dose (0.25 mcg/kg/min) of the remifentanil. These changes are consistent with our prior fNIRS measures of morphine decreasing an evoked noxious heat stimulus; (3) Sex effects—females were observed (without multiple comparison correction) to have an increased response (AUC measure) to ablation in the posterior somatosensory cortex region (presumed nociceptive input from the ablation), an observation not previously evaluated under surgery/anesthesia; and (4) Auditory effects—mFPC response to auditory stimuli were observed to be opposite to nociceptive stimuli (ablation) and this difference between stimulus types was greater in the PL group suggesting that complex subconscious sensory processing, independent of pain may be present under anesthesia.

### Pain effects

We have previously observed and reported a distinct cortical pattern of activation (positive activation in S1 and negative activation or deactivation in the mFPC) following nociceptive stimuli in (1) awake healthy volunteers to painful heat and electrical stimuli [15,20]; (2) in sedated patients undergoing colonoscopy where insufflation of the colon results in correlated grimacing [15]; and (3) in patients undergoing cryo- or electrical ablation for cardiac arrhythmias under general anesthesia [39]. Here, in the patient group receiving PL, we observe the same pattern of activation to RF ablation (see **Fig 3**). Our findings regarding pain effects in PL versus drug group are 2-fold: First, the PL group provides further insight into pain inducing processes resulting in cortical activation under anesthesia. Second, these data reproduce our initial observations in a similar adolescent population with a similar anesthetic technique. In noting our assessment of whether these are painful stimuli, patients under little or no sedation have described catheter ablation as painful, with the majority of patients complaining of chest pain during and after the procedure [40–42]. Pain perception in peripheral and central pathways from the heart are not fully elucidated, although reports for cardiac pain in general are better understood [43,44]. Afferent sensory nerve endings, including thermosensitive receptors, travel via the vagus and nodose ganglion to enter the brain in the nucleus of the tractus solitarius, while sympathetic afferent fibers via the dorsal root ganglion synapse on spinothalamic tract neurons in the dorsal horn of the upper spinal thoracic segments (T1 to T5) [45]. Spinothalamic second-order neurons project to the thalamus, with input relayed not only to the S1 but also to higher-order regions such as the cingulate gyrus, insula, and amygdala, while the third-order neurons project to cortical regions such as the prefrontal cortex. As compared to previous analyses of awake healthy individuals’ responses to heat pain [17], opioids were found to inhibit the cortical response of mFPC and S1 to ablation in the current cohort.

### Opioid effects

As hypothesized, remifentanil (LD or HD) group generally exhibited a diminished ΔHbO response compared with the PL group for all ROIs. Specifically, the mFPC deactivation in response to ablation was found to be significantly diminished in the remifentanil opioid group. Remifentanil was administered as a continuous infusion adjunct to typical anesthetic protocols [46] (see Materials and methods). It is a mu opioid receptor agonist that quickly penetrates the blood brain barrier and indirectly stimulates the descending inhibitory pathways of the brainstem, resulting in reduced transmission of nociceptive afferents to the thalamus [47]. Opioid receptor site maps [48,49] show the greatest binding of mu opioid receptors in the frontolimbic regions, more than somatosensory and temporal regions of the brain—potentially explaining the substantial effect on the mFPC when compared to other ROIs. Prior fMRI studies on the effect of single-infusion remifentanil on pain-related brain activation showed the greatest suppression in the insular cortical activation to pain [50,51]; however, these findings were derived from awake healthy individuals, and the insula is known to be a key region in salience and perception of nociceptive stimuli.

Furthermore, visual observation of the mean hemodynamic response curves (**Fig 3**) in the LD and HD groups indicate a differential hemodynamic response to ablation in the S1 but not the prefrontal cortex of the 2 remifentanil doses—where HD group shows the least variance in response to ablation, whereas LD group appears to initially deactivate and then increase in response to ablation. There was also a notable dose-based effect in ΔHbO measures (Peak_HbO_, Nadir_HbO_, and AUC; **Fig 4B**) where the PL group exhibited the greatest response to ablation, followed by LD and then HD remifentanil groups in the somatosensory region (not statistically significant). These findings suggest that while the dosing may contribute to the extent of diminished cortical responses, it was still insufficient to completely block pain-related activation. Using electrophysiological recordings from cortical regions during nociceptive stimulation in rats under urethane anesthesia, fentanyl (30μg/kg im) inhibited but did not completely eliminate the nociceptive-induced activity [52,53]. Remifentanil like fentanyl may have nonanalgesic effects that include modulation of autonomic activity [54]. Remifentanil may also affect neurovascular coupling [55,56], although this is an unlikely explanation because, while the drug may depress respiratory levels [57], the blood oxygenation levels, and ventilation (under mechanical ventilation) were well maintained within normal limits under the anesthetic regimen deployed in the study. Alternatively, the observed dose dependent response may be explained due to the opioid receptor density in the brain. Some areas (such as S1) may be less susceptible to opioid effects at LDs than other regions of the brain (frontolimbic areas with comparatively higher densities of opioid receptors). An fMRI study using short pulses of CO_2_ to the nasal cavity demonstrated a linear decrease in sensory activation of the brain to an increasing concentration of alfentanil. Interestingly, affective areas of the brain (such as amygdala, anterior insula, and parahippocampal gyrus) showed the greatest attenuation at the lowest dose of alfentanil [58], suggesting that opioids may alter affective processes before pain processing. However, due to the inability of the fNIRS technique to measure deep brain activity, it is unclear how insular or subcortical activity may differ in the conditions tested. Nevertheless, the use of fNIRS and the option to perform whole-brain mapping may be one approach to inform the anesthesiologist of painful events during surgery that may be ongoing or intermittent, even when appropriate analgesics are administered. Even with the current sample size, *n* = 11 in HD, *n* = 12 in LD, and *n* = 9 in PL groups, the differences in effects of remifentanil dosing versus PL were apparent in the average hemodynamic response to ablative procedure. Perhaps, a larger sample size and/or improved postprocessing strategies would more accurately evaluate the dose-dependent effects of remifentanil on the cortex.

### Sex-based differences in pain response

Our study cohort consisted of 16 males and 16 females. Even while accounting for PL or drug dose administered, female patients exhibited a greater response to ablation in the posterior superior somatosensory cortex when compared to male patients. This was significant at *p* < 0.05 (see **Fig 5**), but not significant when evaluated for multiple comparisons. In general, females are at greater risk of developing chronic pain conditions and have greater incidences of postsurgical pain [59]. Sex hormones play a role, at least partly, in the sensitivity and perception of pain (experimental and clinical) [34] and analgesic responses [60] in the 2 sexes, as do emotional and attentive processes [61–63].

### Auditory effects

We used the paradigm described in Materials and methods in an attempt to differentiate pain processing from other subclinical processes that may be present under anesthesia by evaluating cortical changes in the brain in response to auditory stimuli. Our data show that auditory stimuli activates the mFPC, whereas nociceptive stimuli (ablation) deactivates the mFPC. Nociceptive stimuli also appear to result in greater ΔHbO activation measures (Peak_HbO_ and Nadir_HbO_) in mFPC and the posterior SS1 regions in both the groups, although this difference between stimulus types (ablation versus auditory) was greater in PL group than the remifentanil group. In a fMRI study evaluating the brain response to auditory stimulation in healthy volunteers receiving only sevoflurane, Kerssens and colleagues found that ongoing temporal lobe (auditory cortex) activations, as well as in the left frontal cortex, occur at 1% end-tidal sevoflurane [27]. There was no significant auditory-related activation with 2% end-tidal sevoflurane. These authors concluded that residual auditory processing is a reliable phenomenon during light anesthesia (1% end-tidal sevoflurane), but not at deep anesthesia (2% end-tidal sevoflurane). A study with propofol had similar findings in that the primary and association auditory cortices remained responsive to auditory stimuli, albeit at a reduced magnitude (42%), but because higher-level processing for speech and voice was abolished the residual activation in primary regions was insufficient to support formation of memory traces [26]. An electroencephalography study measuring mid-intensity auditory evoked potential to binaural clicks for a duration of 3 minutes reported a decrease in auditory evoked potentials in participants who received remifentanil doses of ≥0.25 mcg/kg/min (irrespective of the level of sedation) [64]. But again, it is unclear how remifentanil may affect other regions of brain networks involved in auditory cognition. Interestingly, there was also a statistical effect of group (whether opioid or PL) on the ΔHbO measures of mFPC, where PL demonstrated a greater response to both stimuli than the remifentanil group. This raises the question of whether remifentanil could affect the cerebrovascular control of specific anatomical regions (such as the prefrontal cortex).

### Strengths and limitations of study

There were a number of caveats that should be noted including the following: (1) Numbers of patients*—*we recruited a total of 41 participants as compared to the initial target of 54 due to the coronavirus pandemic. Out of the 41 participants, 7 were excluded due to poor data quality, and 2 were excluded due to receiving only cryoablations. Even though the number of participants in each group is small and considering the biological variability in pain response, our group-level findings were robust using both multiple ablations as well as the first ablation. Perhaps a larger sample size would better delineate any dose-dependent and sex-related differences in the effect of opioids on nociception during surgery. Also, due to technical difficulties, only 28 of the 32 patients were presented an auditory stimulus during surgery and were included in analysis of the cortical response to pain versus auditory stimulus; (2) Nature of anesthesia paradigm*—*although the mean time from fentanyl administration to the first ablation was 123 ± 40 minutes for all patients (*n* = 30, data unavailable for the remaining 2 patients), the possibility of fentanyl reducing the discriminative effect between the remifentanil groups cannot be excluded; (3) Nature of surgical paradigm—we do not account for interindividual variability in the number of ablations each patient received; however, the response to the first ablation in all participants provided in **S1 Fig** is in agreement with the results obtained from all ablations. Additionally, due to intra- and interparticipant variability in the duration of an ablative procedure, we examined only the first 20 seconds following the beginning of an ablation in all patients. It would be interesting to investigate ongoing changes in ΔHbO to acute versus prolonged ablative procedures as well as any habituation of fNIRS response to repeated nociceptive stimulation. Furthermore, it is currently unclear if variability in medications or procedures such as catheter entry site between patients influences the group-level fNIRS measures; and (4) Technical issues—methodological limitations of fNIRS include limited spatial coverage, superficial penetration depth of the brain, and reduced signal quality in specific brain areas, and individuals due to scalp hair, operating procedure setup, and movement. It is also critical to investigate the relationship between fNIRS measures of nociception with simultaneous physiological and autonomic measures to fully capture the pain state of the brain under anesthesia.

### Implications for future research/clinical practice and public policy

Potential implications of these findings include future research directions, clinical practice, and public policy related to prevention of nociception under surgery.

### Future research directions

Adapting and adopting fNIRS measures in the operating room will require advancements in the following areas: (1) Real-time measures that include evoked and ongoing pain (pain load) over time—there is no gold standard for the quantitative monitoring of nociception intraoperatively, and anesthesiologists titrate analgesics based on autonomic responses via the nociceptive autonomic responses as surrogates; available nociception monitors generate an analgesic index derived from physiologic (mainly autonomic) signals rather than quantifying nociception directly from the central nervous system [65,66]. We have reported on each of these separately [18,67] but not in terms of overall pain load that may contribute to enhanced central sensitization; further research is necessary to define thresholds for intervention; (2) Definition of sensitivity and specificity of fNIRS measures—although the activation’s are context specific, other activations while under anesthesia may need to be evaluated in both awake and anesthetized patients as we have recently reported for painful stimuli [67]; (3) Measures across anesthetic type (e.g., inhalational versus nerve block versus combination) is also necessary; (4) The importance of maintaining optimal analgesia through surgery to inhibit subsequent acute and chronic pain/analgesic use—further work is required to quantitate the relationship of perioperative pain to sensitization and the development of chronic pain; as such, determination of presurgical factors (brain state and physiological/autonomic activity) influence the measurement of pain during surgery; and (5) Technical development—if shown to be useful, the technology will need to be developed into a format that can be used at the bedside/in the operating room.

### Clinical practice

We have recently reviewed fNIRS as a tool to measure nociception, pain and analgesia that may be translated to clinical practice [39]; ongoing nociception/pain may be present with repeated afferent painful barrage or ongoing inflammatory-induced activation of damaged nerves (nociceptors) and sensitized brain systems (central sensitization) [3,68]. The latter has implications for the development of chronic pain [69]; fNIRS has the potential to be developed into a monitor for the direct measurement of nociception in patients under general anesthesia. There is no gold standard for the quantitative monitoring of nociception intraoperatively, and anesthesiologists titrate analgesics based on autonomic responses via the nociceptive autonomic medullary circuit as surrogates. Currently available nociception monitors generate an analgesic index derived from physiologic (mainly autonomic) signals rather than quantifying nociception from the central nervous system directly [65,66]. Having an objective measure for nociception/pain may help manage individuals who cannot communicate but may have pain during the perioperative period (e.g., newborns [70]) and individuals incapacitated by stroke or have Alzheimer disease. Additionally, the technology may eventually be used for assessing ongoing pain in patients as our recent results suggest may be possible with fNIRS [67].

### Public policy

Surgery is a common medical procedure with some 319 million surgeries performed in 2012 [71] globally and some 28.6 million ambulatory surgeries performed each year in the USA alone [72]. Although uncommon, surgical awareness [73,74], where patients may feel “auditory perception, the feeling of motor function lost, pain, helplessness, anxiety, panic, impending death,” is reported [74]. Currently, there is no good monitor for objective monitoring of surgical awareness. fNIRS can be adapted to measure such changes as defined in the approach suggested for auditory measures or commands in this paper. A much larger public healthcare problem relates to (1) opioid use disorder following surgery if opioids are used to limit postsurgical pain [75,76]; and (2) the evolution of surgically induced chronic pain estimated to be around 30% of all surgical patients [69,77,78], both of which cost society billions of dollars and patients and their families significant suffering [79,80]. Having an objective measure for intraoperative nociception may allow for true evaluation of current and future drugs or procedures to enhance preventive analgesia and limit chronic postsurgical pain [81].

## Conclusions

The ability to evaluate nociceptive processing during anesthesia would allow for improved evaluation of surgically induced pain during surgery. Evoked (e.g., incision) or ongoing (e.g., inflammatory and other nociceptive molecules released by surgical trauma) painful stimuli result in a nociceptive barrage that is not easily evaluated clinically. Having an objective marker of nociception would likely transform the surgical/anesthesia event along the lines pulse oximetry changed the evaluation of a patient’s oxygen levels in a way that allows for immediate evaluation and intervention. While further work will be required to define the sensitivity and specificity of the use of fNIRS as an objective marker of nociceptive activity under anesthesia, the present study provides further information toward this goal.

## Supporting information

S1 Checklist CONSORT Checklist. CONSORT, Consolidated Standards of Reporting Trials.
(DOC)

S2 Checklist CONSERVE Checklist. CONSERVE, CONSORT and SPIRIT Extension for Randomized Clinical Trials in Extenuating Circumstances.
(DOCX)

S1 Fig Group-averaged hemodynamic response in the 6 ROIs to the first ablation event. ROI, region of interest.
(DOCX)

S2 Fig Group-averaged hemodynamic response to ablation in all 24 channels. (DOCX)

S3 Fig Sex-related effects in response to ablation in male versus female patients >14 years of age during general anesthesia. (DOCX)

S4 Fig AUC measures following painful versus auditory stimuli under general anesthesia. AUC, area under the ΔHbO curve.
(DOCX)

S1 Table Results of independent sample *t* test comparing cortical activation measures during ablation between male and female patients. (DOCX)

S1 Protocol Assessing the cortical response to noxious and auditory stimuli using near infrared spectroscopy in participants under general anesthesia. (PDF)

## Abbreviations

ANOVA: analysis of covariance
Ant. SS1: anterior superior S1
AUC: area under the ΔHbO curve
BIS: bispectral index
CI: confidence interval
CONSERVE: CONSORT and SPIRIT Extension for Randomized Clinical Trials in Extenuating Circumstances
CONSORT: Consolidated Standards of Reporting Trials
FDR: false discovery rate
fMRI: functional magnetic resonance imaging
fNIRS: functional near-infrared spectroscopy
HD: high-dose
Inf. lPFC: inferior lateral prefrontal cortex
Inf. mFPC: inferior medial frontopolar cortex
lPFC: lateral prefrontal cortex
IRB: Institutional Review Board
ITT: intention-to-treat
LD: low-dose
MD: mean difference
mFPC: medial frontopolar cortex
Peak_HbO_: Peak ΔHbO
PL: placebo
Pos. SS1: posterior superior S1
RF: radiofrequency
ROI: region of interest
S1: primary somatosensory cortex
SMD: standardized mean difference
Sup. lPFC: superior lateral prefrontal cortex
Sup. mFPC: superior medial frontopolar cortex

## References

[1] Akeju O, Brown EN. Neural oscillations demonstrate that general anesthesia and sedative states are neurophysiologically distinct from sleep. Curr Opin Neurobiol. 2017;44:178–85. doi: 10.1016/j.conb.2017.04.011 28544930 PMC5520989

[2] Kehlet H, Jensen TS, Woolf CJ. Persistent postsurgical pain: risk factors and prevention. Lancet. 2006;367(9522):1618–25. doi: 10.1016/S0140-6736(06)68700-X 16698416

[3] Woolf CJ. Central sensitization: implications for the diagnosis and treatment of pain. Pain. 2011;152(3 Suppl):S2–15. doi: 10.1016/j.pain.2010.09.030 20961685 PMC3268359

[4] Brown EN, Pavone KJ, Naranjo M. Multimodal General Anesthesia: Theory and Practice. Anesth Analg. 2018;127(5):1246–58. doi: 10.1213/ANE.0000000000003668 30252709 PMC6203428

[5] Liang X, Liu R, Chen C, Ji F, Li T. Opioid System Modulates the Immune Function: A Review. Transl Perioper Pain Med. 2016;1(1):5–13. 26985446 PMC4790459

[6] Stein C, Kuchler S. Non-analgesic effects of opioids: peripheral opioid effects on inflammation and wound healing. Curr Pharm Des. 2012;18(37):6053–69. doi: 10.2174/138161212803582513 22747536

[7] Kanjhan R. Opioids and pain. Clin Exp Pharmacol Physiol. 1995;22(6–7):397–403. doi: 10.1111/j.1440-1681.1995.tb02029.x 8582088

[8] Fornasari D. Pain mechanisms in patients with chronic pain. Clin Drug Investig. 2012;32(Suppl 1):45–52. 10.2165/11630070-000000000-00000 22356223

[9] Damien J, Colloca L, Bellei-Rodriguez CE, Marchand S. Pain Modulation: From Conditioned Pain Modulation to Placebo and Nocebo Effects in Experimental and Clinical Pain. Int Rev Neurobiol. 2018;139:255–96. doi: 10.1016/bs.irn.2018.07.024 30146050 PMC6175288

[10] Brown EN, Lydic R, Schiff ND. General anesthesia, sleep, and coma. N Engl J Med. 2010;363(27):2638–50. doi: 10.1056/NEJMra0808281 21190458 PMC3162622

[11] Mastronardi P, Dellacasa P. Observational study on the use of remifentanil in general anesthesia. Drug utilisation research. Minerva Anestesiol. 2004;70(7–8):605–16. 15252372

[12] Hah JM, Bateman BT, Ratliff J, Curtin C, Sun E. Chronic Opioid Use After Surgery: Implications for Perioperative Management in the Face of the Opioid Epidemic. Anesth Analg. 2017;125(5):1733–40. doi: 10.1213/ANE.0000000000002458 29049117 PMC6119469

[13] Liu SS, Strodtbeck WM, Richman JM, Wu CL. A comparison of regional versus general anesthesia for ambulatory anesthesia: a meta-analysis of randomized controlled trials. Anesth Analg. 2005;101(6):1634–42. doi: 10.1213/01.ANE.0000180829.70036.4F 16301234

[14] Richman JM, Liu SS, Courpas G, Wong R, Rowlingson AJ, McGready J, et al. Does continuous peripheral nerve block provide superior pain control to opioids? A meta-analysis. Anesth Analg. 2006;102(1):248–57. doi: 10.1213/01.ANE.0000181289.09675.7D 16368838

[15] Becerra L, Aasted CM, Boas DA, George E, Yucel MA, Kussman BD, et al. Brain measures of nociception using near-infrared spectroscopy in patients undergoing routine screening colonoscopy. Pain. 2016;157(4):840–8. doi: 10.1097/j.pain.0000000000000446 26645550 PMC4794375

[16] Kotlyar BI, Timofeeva NO. State of the brain as a systemic neurophysiological mechanism of the conditioned reflex. Neurosci Behav Physiol. 1989;19(1):1–6. doi: 10.1007/BF01148403 2664550

[17] Peng K, Yucel MA, Steele SC, Bittner EA, Aasted CM, Hoeft MA, et al. Morphine Attenuates fNIRS Signal Associated With Painful Stimuli in the Medial Frontopolar Cortex (medial BA 10). Front Hum Neurosci. 2018;12:394. doi: 10.3389/fnhum.2018.00394 30349466 PMC6186992

[18] Kussman BD, Aasted CM, Yucel MA, Steele SC, Alexander ME, Boas DA, et al. Capturing Pain in the Cortex during General Anesthesia: Near Infrared Spectroscopy Measures in Patients Undergoing Catheter Ablation of Arrhythmias. PLoS ONE. 2016;11(7):e0158975. doi: 10.1371/journal.pone.0158975 27415436 PMC4944937

[19] Scott LJ, Perry CM. Remifentanil: a review of its use during the induction and maintenance of general anaesthesia. Drugs. 2005;65(13):1793–823. doi: 10.2165/00003495-200565130-00007 16114980

[20] Yucel MA, Aasted CM, Petkov MP, Borsook D, Boas DA, Becerra L. Specificity of hemodynamic brain responses to painful stimuli: a functional near-infrared spectroscopy study. Sci Rep. 2015;5:9469. doi: 10.1038/srep09469 25820289 PMC4377554

[21] Peng K, Steele SC, Becerra L, Borsook D. Brodmann area 10: Collating, integrating and high level processing of nociception and pain. Prog Neurobiol. 2018;161:1–22. doi: 10.1016/j.pneurobio.2017.11.004 29199137 PMC5826795

[22] Araldi D, Khomula EV, Ferrari LF, Levine JD. Fentanyl Induces Rapid Onset Hyperalgesic Priming: Type I at Peripheral and Type II at Central Nociceptor Terminals. J Neurosci. 2018;38(9):2226–45. doi: 10.1523/JNEUROSCI.3476-17.2018 29431655 PMC5830512

[23] Hogan D, Baker AL, Moron JA, Carlton SM. Systemic morphine treatment induces changes in firing patterns and responses of nociceptive afferent fibers in mouse glabrous skin. Pain. 2013;154(11):2297–309. doi: 10.1016/j.pain.2013.05.033 23711478 PMC3806901

[24] Rajan R, Dubaj V, Reser DH, Rosa MG. Auditory cortex of the marmoset monkey—complex responses to tones and vocalizations under opiate anaesthesia in core and belt areas. Eur J Neurosci. 2013;37(6):924–41. doi: 10.1111/ejn.12092 23278961

[25] Oroei M, Peyvandi AA, Mokhtarinejad F. Opioid Drugs and Sensorineural Hearing Loss. Addict Health. 2018;10(1):64–6. doi: 10.22122/ahj.v10i1.560 30627386 PMC6312560

[26] Plourde G, Belin P, Chartrand D, Fiset P, Backman SB, Xie G, et al. Cortical processing of complex auditory stimuli during alterations of consciousness with the general anesthetic propofol. Anesthesiology. 2006;104(3):448–57. doi: 10.1097/00000542-200603000-00011 16508391

[27] Kerssens C, Hamann S, Peltier S, Hu XP, Byas-Smith MG, Sebel PS. Attenuated brain response to auditory word stimulation with sevoflurane: a functional magnetic resonance imaging study in humans. Anesthesiology. 2005;103(1):11–9. doi: 10.1097/00000542-200507000-00006 15983451

[28] Brigadoi S, Cooper RJ. How short is short? Optimum source-detector distance for short-separation channels in functional near-infrared spectroscopy. Neurophotonics. 2015;2(2):025005. doi: 10.1117/1.NPh.2.2.025005 26158009 PMC4478880

[29] Molavi B, Dumont GA. Wavelet-based motion artifact removal for functional near-infrared spectroscopy. Physiol Meas. 2012;33(2):259–70. doi: 10.1088/0967-3334/33/2/259 22273765

[30] Brigadoi S, Ceccherini L, Cutini S, Scarpa F, Scatturin P, Selb J, et al. Motion artifacts in functional near-infrared spectroscopy: a comparison of motion correction techniques applied to real cognitive data. Neuroimage. 2014;85. PubMed PMID: Pt 1:181–91. doi: 10.1016/j.neuroimage.2013.04.082 23639260 PMC3762942

[31] Huppert TJ, Diamond SG, Franceschini MA, Boas DA. HomER: a review of time-series analysis methods for near-infrared spectroscopy of the brain. Appl Optics. 2009;48(10):D280–98. doi: 10.1364/ao.48.00d280 19340120 PMC2761652

[32] Benjamini Y, Hochberg Y. Controlling the False Discovery Rate: A Practical and Powerful Approach to Multiple Testing. J R Stat Soc B Methodol. 1995;57(1):289–300.

[33] Benjamini Y, Yekutieli D. False Discovery Rate–Adjusted Multiple Confidence Intervals for Selected Parameters. J Am Stat Assoc. 2005;100(469):71–81.

[34] Mogil JS. Qualitative sex differences in pain processing: emerging evidence of a biased literature. Nat Rev Neurosci. 2020;21(7):353–65. doi: 10.1038/s41583-020-0310-6 32440016

[35] Chen YH, Lin H, Xie CL, Zhang XT, Li YG. Efficacy comparison between cryoablation and radiofrequency ablation for patients with cavotricuspid valve isthmus dependent atrial flutter: a meta-analysis. Sci Rep. 2015;5:10910. doi: 10.1038/srep10910 26039980 PMC4454189

[36] Bravo L, Atienza F, Eidelman G, Avila P, Pelliza M, Castellanos E, et al. Safety and efficacy of cryoablation vs. radiofrequency ablation of septal accessory pathways: systematic review of the literature and meta-analyses. Europace. 2018;20(8):1334–42. doi: 10.1093/europace/eux269 29036312

[37] Hanninen M, Yeung-Lai-Wah N, Massel D, Gula LJ, Skanes AC, Yee R, et al. Cryoablation versus RF ablation for AVNRT: A meta-analysis and systematic review. J Cardiovasc Electrophysiol. 2013;24(12):1354–60. doi: 10.1111/jce.12247 24016223

[38] Norgaard MW, Pedersen PU, Bjerrum M. Understanding how patients use visualization during ablation of atrial fibrillation in reducing their experience of pain, anxiety, consumption of pain medication and procedure length: Integrating quantitative and qualitative results. Appl Nurs Res. 2018;39:229–40. doi: 10.1016/j.apnr.2017.11.026 29422164

[39] Karunakaran KD, Peng K, Berry D, Green S, Labadie R, Kussman B, et al. NIRS measures in pain and analgesia: Fundamentals, features, and function. Neurosci Biobehav Rev. 2021;120:335–53. doi: 10.1016/j.neubiorev.2020.10.023 33159918

[40] Aryana A, Heist EK, D’Avila A, Holmvang G, Chevalier J, Ruskin JN, et al. Pain and anatomical locations of radiofrequency ablation as predictors of esophageal temperature rise during pulmonary vein isolation. J Cardiovasc Electrophysiol. 2008;19(1):32–8. doi: 10.1111/j.1540-8167.2007.00975.x 17900251

[41] Alaeddini J, Wood MA, Parvez B, Pathak V, Wong KA, Ellenbogen KA. Site localization and characterization of pain during radiofrequency ablation of the pulmonary veins. Pacing Clin Electrophysiol. 2007;30(10):1210–4. doi: 10.1111/j.1540-8159.2007.00842.x 17897123

[42] Bode K, Breithardt OA, Kreuzhuber M, Mende M, Sommer P, Richter S, et al. Patient discomfort following catheter ablation and rhythm device surgery. Europace. 2015;17(7):1129–35. doi: 10.1093/europace/euu325 25488958

[43] Foreman RD, Garrett KM, Blair RW. Mechanisms of cardiac pain. Compr Physiol. 2015;5(2):929–60. doi: 10.1002/cphy.c140032 25880519

[44] Rosen SD, Camici PG. The brain-heart axis in the perception of cardiac pain: the elusive link between ischaemia and pain. Ann Med. 2000;32(5):350–64. doi: 10.3109/07853890008995938 10949067

[45] Premkumar LS, Raisinghani M. Nociceptors in cardiovascular functions: complex interplay as a result of cyclooxygenase inhibition. Mol Pain. 2006;2:26. doi: 10.1186/1744-8069-2-26 16916451 PMC1563450

[46] Beers R, Camporesi E. Remifentanil update: clinical science and utility. CNS Drugs. 2004;18(15):1085–104. doi: 10.2165/00023210-200418150-00004 15581380

[47] Pathan H, Williams J. Basic opioid pharmacology: an update. Br J Pain. 2012;6(1):11–6. doi: 10.1177/2049463712438493 26516461 PMC4590096

[48] Rabiner EA, Beaver J, Makwana A, Searle G, Long C, Nathan PJ, et al. Pharmacological differentiation of opioid receptor antagonists by molecular and functional imaging of target occupancy and food reward-related brain activation in humans. Mol Psychiatry. 2011;16(8):826–35, 785. doi: 10.1038/mp.2011.29 21502953 PMC3142667

[49] Martin M, Hurley RA, Taber KH. Is opiate addiction associated with longstanding neurobiological changes? J Neuropsychiatry Clin Neurosci. 2007;19(3):242–8. doi: 10.1176/jnp.2007.19.3.242 17827409

[50] Wise RG, Williams P, Tracey I. Using fMRI to quantify the time dependence of remifentanil analgesia in the human brain. Neuropsychopharmacology. 2004;29(3):626–35. doi: 10.1038/sj.npp.1300364 14679387

[51] Wise RG, Rogers R, Painter D, Bantick S, Ploghaus A, Williams P, et al. Combining fMRI with a pharmacokinetic model to determine which brain areas activated by painful stimulation are specifically modulated by remifentanil. Neuroimage. 2002;16(4):999–1014. doi: 10.1006/nimg.2002.1146 12202088

[52] Peng YZ, Li XX, Wang YW. Effects of Parecoxib and Fentanyl on nociception-induced cortical activity. Mol Pain. 2010;6:3. doi: 10.1186/1744-8069-6-3 20089200 PMC2819047

[53] Kibaly C, Xu C, Cahill CM, Evans CJ, Law PY. Non-nociceptive roles of opioids in the CNS: opioids’ effects on neurogenesis, learning, memory and affect. Nat Rev Neurosci. 2019;20(1):5–18. doi: 10.1038/s41583-018-0092-2 30518959 PMC6736526

[54] Feld J, Hoffman WE, Paisansathan C, Park H, Ananda RC. Autonomic activity during dexmedetomidine or fentanyl infusion with desflurane anesthesia. J Clin Anesth. 2007;19(1):30–6. doi: 10.1016/j.jclinane.2006.05.019 17321924

[55] Pattinson KT, Wise RG. Imaging the Respiratory Effects of Opioids in the Human Brain. Adv Exp Med Biol. 2016;903:145–56. doi: 10.1007/978-1-4899-7678-9_10 27343094

[56] Pattinson KT, Rogers R, Mayhew SD, Tracey I, Wise RG. Pharmacological FMRI: measuring opioid effects on the BOLD response to hypercapnia. J Cereb Blood Flow Metab. 2007;27(2):414–23. doi: 10.1038/sj.jcbfm.9600347 16736039

[57] Kiyatkin EA. Respiratory depression and brain hypoxia induced by opioid drugs: Morphine, oxycodone, heroin, and fentanyl. Neuropharmacology. 2019;151:219–26. doi: 10.1016/j.neuropharm.2019.02.008 30735692 PMC6500744

[58] Oertel BG, Preibisch C, Wallenhorst T, Hummel T, Geisslinger G, Lanfermann H, et al. Differential opioid action on sensory and affective cerebral pain processing. Clin Pharmacol Ther. 2008;83(4):577–88. doi: 10.1038/sj.clpt.6100441 18030306

[59] Fillingim RB, King CD, Ribeiro-Dasilva MC, Rahim-Williams B, Riley JL 3rd. Sex, gender, and pain: a review of recent clinical and experimental findings. J Pain. 2009;10(5):447–85. doi: 10.1016/j.jpain.2008.12.001 19411059 PMC2677686

[60] Fillingim RB. Sex differences in analgesic responses: evidence from experimental pain models. Eur J Anaesthesiol Suppl. 2002;26:16–24. doi: 10.1097/00003643-200219261-00004 12512212

[61] Goffaux P, Michaud K, Gaudreau J, Chalaye P, Rainville P, Marchand S. Sex differences in perceived pain are affected by an anxious brain. Pain. 2011;152(9):2065–73. doi: 10.1016/j.pain.2011.05.002 21665365

[62] Kano M, Farmer AD, Aziz Q, Giampietro VP, Brammer MJ, Williams SC, et al. Sex differences in brain response to anticipated and experienced visceral pain in healthy subjects. Am J Physiol Gastrointest Liver Physiol. 2013;304(8):G687–99. doi: 10.1152/ajpgi.00385.2012 23392235 PMC3625873

[63] Popovich C, Dockstader C, Cheyne D, Tannock R. Sex differences in sensorimotor mu rhythms during selective attentional processing. Neuropsychologia. 2010;48(14):4102–10. doi: 10.1016/j.neuropsychologia.2010.10.016 20951711

[64] Supp GG, Higgen FL, Hipp JF, Engel AK, Siegel M. Mid-Latency Auditory Evoked Potentials Differentially Predict Sedation and Drug Level Under Opioid and Hypnotic Agents. Front Pharmacol. 2018;9:1427. doi: 10.3389/fphar.2018.01427 30564126 PMC6288227

[65] Anderson TA. Intraoperative Analgesia-Nociception Monitors: Where We Are and Where We Want To Be. Anesth Analg. 2020;130(5):1261–3. doi: 10.1213/ANE.0000000000004473 32287133

[66] Funcke S, Pinnschmidt HO, Wesseler S, Brinkmann C, Beyer B, Jazbutyte V, et al. Guiding Opioid Administration by 3 Different Analgesia Nociception Monitoring Indices During General Anesthesia Alters Intraoperative Sufentanil Consumption and Stress Hormone Release: A Randomized Controlled Pilot Study. Anesth Analg. 2020;130(5):1264–73. doi: 10.1213/ANE.0000000000004388 31517677

[67] Peng K, Deepti Karunakaran K, Lee A, Gomez-Morad A, Labadie R, Mizrahi-Arnaud A, et al. Rhythmic Change of Cortical Hemodynamic Signals Associated with Ongoing Nociception in Awake and Anesthetized Individuals: An Exploratory Functional Near Infrared Spectroscopy Study. Anesthesiology. 2021;135(5):877–92. doi: 10.1097/ALN.0000000000003986 34610092 PMC8511051

[68] Juhl GI, Jensen TS, Norholt SE, Svensson P. Central sensitization phenomena after third molar surgery: a quantitative sensory testing study. Eur J Pain. 2008;12(1):116–27. doi: 10.1016/j.ejpain.2007.04.002 17553713

[69] Borsook D, Kussman BD, George E, Becerra LR, Burke DW. Surgically induced neuropathic pain: understanding the perioperative process. Ann Surg. 2013;257(3):403–12. doi: 10.1097/SLA.0b013e3182701a7b 23059501 PMC3546123

[70] Eriksson M, Campbell-Yeo M. Assessment of pain in newborn infants. Semin Fetal Neonatal Med. 2019;24(4):101003. doi: 10.1016/j.siny.2019.04.003 30987943

[71] Weiser TG, Haynes AB, Molina G, Lipsitz SR, Esquivel MM, Uribe-Leitz T, et al. Size and distribution of the global volume of surgery in 2012. Bull World Health Organ. 2016;94(3):201–9F. doi: 10.2471/BLT.15.159293 26966331 PMC4773932

[72] Hall MJ, Schwartzman A, Zhang J, Liu X. Ambulatory Surgery Data From Hospitals and Ambulatory Surgery Centers: United States, 2010. Natl Health Stat Rep. 2017;102:1–15. 28256998

[73] Bischoff P, Rundshagen I. Awareness under general anesthesia. Dtsch Arztebl Int. 2011;108(1–2):1–7. doi: 10.3238/arztebl.2011.0001 21285993 PMC3026393

[74] Radovanovic D, Radovanovic Z. Awareness during general anaesthesia—implications of explicit intraoperative recall. Eur Rev Med Pharmacol Sci. 2011;15(9):1085–9. 22013733

[75] Brummett CM, Waljee JF, Goesling J, Moser S, Lin P, Englesbe MJ, et al. New Persistent Opioid Use After Minor and Major Surgical Procedures in US Adults. JAMA Surg. 2017;152(6):e170504. doi: 10.1001/jamasurg.2017.0504 28403427 PMC7050825

[76] Clarke H, Soneji N, Ko DT, Yun L, Wijeysundera DN. Rates and risk factors for prolonged opioid use after major surgery: population based cohort study. BMJ. 2014;348:g1251. doi: 10.1136/bmj.g1251 24519537 PMC3921439

[77] Humble SR, Dalton AJ, Li L. A systematic review of therapeutic interventions to reduce acute and chronic post-surgical pain after amputation, thoracotomy or mastectomy. Eur J Pain. 2015;19(4):451–65. doi: 10.1002/ejp.567 25088289 PMC4405062

[78] Schnabel A, Pogatzki-Zahn E. Predictors of chronic pain following surgery. What do we know? Schmerz. 2010;24(5):517–31. PubMed PMID: quiz 32–3. doi: 10.1007/s00482-010-0932-0 20798959

[79] Bruce J, Quinlan J. Chronic Post Surgical Pain. Rev Pain. 2011;5(3):23–9. doi: 10.1177/204946371100500306 26526062 PMC4590073

[80] Guertin JR, Page MG, Tarride JE, Talbot D, Watt-Watson J, Choiniere M. Just how much does it cost? A cost study of chronic pain following cardiac surgery. J Pain Res. 2018;11:2741–59. doi: 10.2147/JPR.S175090 30519078 PMC6235323

[81] Clarke H, Poon M, Weinrib A, Katznelson R, Wentlandt K, Katz J. Preventive analgesia and novel strategies for the prevention of chronic post-surgical pain. Drugs. 2015;75(4):339–51. doi: 10.1007/s40265-015-0365-2 25752774

