# Supplementary material for: Brain-based measures of nociception during general anesthesia with remifentanil: A randomized controlled trial
Source: PLoS Med. 2022 Apr 22;19(4):e1003965. doi: 10.1371/journal.pmed.1003965 (PMC9075662; doi:10.1371/journal.pmed.1003965)
Supplement: S1 Checklist — CONSORT, Consolidated Standards of Reporting Trials. (DOC) [file pmed.1003965.s001.doc]

CONSORT 2010 checklist of information to include when reporting a randomised trial*

| Section/Topic | Item No | Checklist item | Reported on page No |
| --- | --- | --- | --- |
| Title and abstract | | | |
|  | 1a | Identification as a randomised trial in the title | First lines of title page |
|  | 1b | Structured summary of trial design, methods, results, and conclusions (for specific guidance see CONSORT for abstracts) | Abstract |
| Introduction | | | |
| Background and objectives | 2a | Scientific background and explanation of rationale | First, second and third paragraphs of Introduction |
|  | 2b | Specific objectives or hypotheses | Fourth and fifth paragraphs of Introduction |
| Methods | | | |
| Trial design | 3a | Description of trial design (such as parallel, factorial) including allocation ratio | Second paragraph of Methods section |
|  | 3b | Important changes to methods after trial commencement (such as eligibility criteria), with reasons | N/A |
| Participants | 4a | Eligibility criteria for participants | First paragraph of Methods section |
|  | 4b | Settings and locations where the data were collected | First paragraph of Methods section |
| Interventions | 5 | The interventions for each group with sufficient details to allow replication, including how and when they were actually administered | Fifth paragraph of Methods section |
| Outcomes | 6a | Completely defined pre-specified primary and secondary outcome measures, including how and when they were assessed | Methods section of Abstract and |
|  | 6b | Any changes to trial outcomes after the trial commenced, with reasons | Ninth and Eleventh paragraph of Methods section |
| Sample size | 7a | How sample size was determined | Ninth paragraph in Methods section |
|  | 7b | When applicable, explanation of any interim analyses and stopping guidelines | Third paragraph of Methods section |
| Randomisation: |  |  |  |
| Sequence generation | 8a | Method used to generate the random allocation sequence | Second paragraph of Methods section |
|  | 8b | Type of randomisation; details of any restriction (such as blocking and block size) | Second paragraph of Methods section |
| Allocation concealment mechanism | 9 | Mechanism used to implement the random allocation sequence (such as sequentially numbered containers), describing any steps taken to conceal the sequence until interventions were assigned | Second paragraph of Methods section |
| Implementation | 10 | Who generated the random allocation sequence, who enrolled participants, and who assigned participants to interventions | First and second paragraphs of Methods |
| Blinding | 11a | If done, who was blinded after assignment to interventions (for example, participants, care providers, those assessing outcomes) and how | Methods section of abstract and Methods section paragraph 2 |
|  | 11b | If relevant, description of the similarity of interventions | N/A |
| Statistical methods | 12a | Statistical methods used to compare groups for primary and secondary outcomes | Tenth paragraph of Methods section |
|  | 12b | Methods for additional analyses, such as subgroup analyses and adjusted analyses | Eleventh and twelfth paragraphs in Methods |
| Results | | | |
| Participant flow (a diagram is strongly recommended) | 13a | For each group, the numbers of participants who were randomly assigned, received intended treatment, and were analysed for the primary outcome | First paragraph of Results section |
|  | 13b | For each group, losses and exclusions after randomisation, together with reasons | First paragraph of Results section |
| Recruitment | 14a | Dates defining the periods of recruitment and follow-up | First paragraph of Results section |
|  | 14b | Why the trial ended or was stopped | First and third paragraph of Methods and Findings. |
| Baseline data | 15 | A table showing baseline demographic and clinical characteristics for each group | First paragraph of Results section |
| Numbers analysed | 16 | For each group, number of participants (denominator) included in each analysis and whether the analysis was by original assigned groups | First paragraph of Results section |
| Outcomes and estimation | 17a | For each primary and secondary outcome, results for each group, and the estimated effect size and its precision (such as 95% confidence interval) | Third and forth paragraph of Results |
|  | 17b | For binary outcomes, presentation of both absolute and relative effect sizes is recommended | N/A |
| Ancillary analyses | 18 | Results of any other analyses performed, including subgroup analyses and adjusted analyses, distinguishing pre-specified from exploratory | Paragrsaphs five to ten of Results section |
| Harms | 19 | All important harms or unintended effects in each group (for specific guidance see CONSORT for harms) | Second paragraph of Results section |
| Discussion | | | |
| Limitations | 20 | Trial limitations, addressing sources of potential bias, imprecision, and, if relevant, multiplicity of analyses | Eighth paragraph of Discussion |
| Generalisability | 21 | Generalisability (external validity, applicability) of the trial findings | Nineth, tenth, eleventh paragraphs of Discussion |
| Interpretation | 22 | Interpretation consistent with results, balancing benefits and harms, and considering other relevant evidence | Paragraphs four to seven of Discussion |
| Other information | | |  |
| Registration | 23 | Registration number and name of trial registry | Methods section of Abstract, and first paragraph of Methods |
| Protocol | 24 | Where the full trial protocol can be accessed, if available | First paragraph of Methods section |
| Funding | 25 | Sources of funding and other support (such as supply of drugs), role of funders | Funding Statement |

*We strongly recommend reading this statement in conjunction with the CONSORT 2010 Explanation and Elaboration for important clarifications on all the items. If relevant, we also recommend reading CONSORT extensions for cluster randomised trials, non-inferiority and equivalence trials, non-pharmacological treatments, herbal interventions, and pragmatic trials. Additional extensions are forthcoming: for those and for up to date references relevant to this checklist, see [www.consort-statement.org](http://www.consort-statement.org/).
