# Supplementary material for: Brain-based measures of nociception during general anesthesia with remifentanil: A randomized controlled trial
Source: PLoS Med. 2022 Apr 22;19(4):e1003965. doi: 10.1371/journal.pmed.1003965 (PMC9075662; doi:10.1371/journal.pmed.1003965)
Supplement: S2 Checklist — CONSERVE, CONSORT and SPIRIT Extension for Randomized Clinical Trials in Extenuating Circumstances. (DOCX) [file pmed.1003965.s002.docx]

CONSERVE Checklists

Use CONSERVE-CONSORT for completed trial reports and CONSERVE-SPIRIT for trial protocols.

| **CONSERVE-CONSORT Extension: [DATE]** | | | | | |
| --- | --- | --- | --- | --- | --- |
| **Item** | **Item Title** | **Description** | | | **Page No.** |
| I. | Extenuating Circumstances | Describe the circumstances and how they constitute extenuating circumstances. | | | First and third paragraph of Materials and Methods |
| II. | Important Modifications | a. Describe how the modifications are important modifications. | | | First and third paragraph of Materials and Methods |
|  |  | b. Describe the impacts and mitigating strategies, including their rationale and implications for the trial. | | | (see below) |
|  |  | c. Provide a modification timeline. | | | Third paragraph of Material and Methods |
| III. | Responsible Parties | State who planned, reviewed and approved the modifications. | | | Third paragraph of Materials and Methods |
| IV. | Interim data | If modifications were informed by trial data, describe how the interim data were used, including whether they were examined by study group, and whether the individuals reviewing the data were blinded to the treatment allocation. | | | Third paragraph of Materials and Methods |
| **CONSORT Number and Item** | | For each row, if important modifications occurred check “direct impact” and/or “mitigating strategy” and describe the changes in the trial manuscript or supplement. Check “no change” for items that are unaffected in the extenuating circumstance. | | | **Page No.** |
|  |  | **No Change** | **Impact*** | **Mitigating Strategy**** |  |
| 1 | Title and abstract | X |  |  |  |
| 2 | Introduction | X |  |  |  |
| 3 | Methods: Trial Design | X |  |  |  |
| 4 | Methods: Participants |  |  |  |  |
| 5 | Methods: Interventions | X |  |  |  |
| 6 | Methods: Outcomes | X |  |  |  |
| 7 | Methods: Sample Size |  | X |  | First and third paragraph of Materials and Methods |
| 8-10 | Methods: Randomisation | X |  |  |  |
| 11 | Methods: Blinding | X |  |  |  |
| 12 | Methods: Statistical methods | X |  |  |  |
| 13 | Results: Participant flow | X |  |  |  |
| 14 | Results: Recruitment |  | X |  | First paragraph of Materials and Methods |
| 15 | Results: Baseline data | X |  |  |  |
| 16 | Results: Numbers analysed |  | X |  | First paragraph of Results |
| 17 | Results: Outcomes and estimation | X |  |  |  |
| 18 | Results: Ancillary analyses | X |  |  |  |
| 19 | Results: Harms | X |  |  |  |
| 20 | Discussion: Limitations |  | X |  | Eighth paragraph of Results |
| 21 | Discussion: Generalisability | X |  |  |  |
| 22 | Other information: Registration | X |  |  |  |
| 23 | Other information: Protocol | X |  |  |  |
| 24 | Other information: Funding | X |  |  |  |
