# Supplementary material for: Brain-based measures of nociception during general anesthesia with remifentanil: A randomized controlled trial
Source: PLoS Med. 2022 Apr 22;19(4):e1003965. doi: 10.1371/journal.pmed.1003965 (PMC9075662; doi:10.1371/journal.pmed.1003965)
Supplement: S1 Fig — ROI, region of interest. (DOCX) [file pmed.1003965.s003.docx]

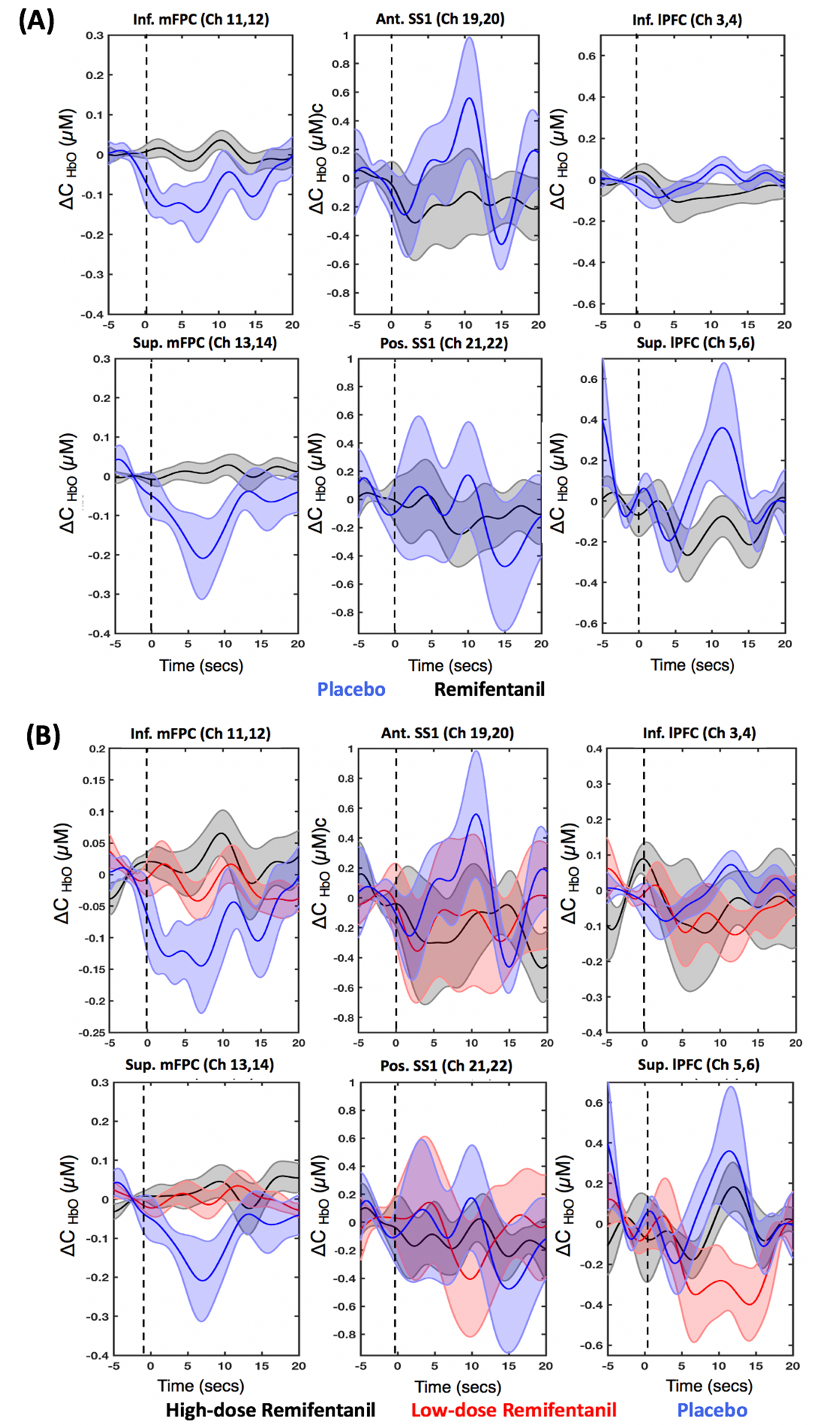


**S1 Fig:** **Group-Averaged Hemodynamic Response in the Six Regions of Interest to the First Ablation Event:** A: Remifentanil (HD + LD=black) and Placebo groups (blue); B: Remifentanil subgroups (HD=black; LD=red) and placebo (blue) group. The dotted black line indicates the start of ablation. The shaded area represents the standard error of mean. **Abbreviations:** Inf. mPFC, inferior medial frontopolar cortex; Sup. mPFC, superior medial frontopolar cortex; Ant. SS1, anterior superior somatosensory cortex; Pos. SS1, posterior superior somatosensory cortex; Inf. lPFC, inferior lateral prefrontal cortex; and Sup. lPFC, superior lateral prefrontal cortex.
