# Supplementary material for: Brain-based measures of nociception during general anesthesia with remifentanil: A randomized controlled trial
Source: PLoS Med. 2022 Apr 22;19(4):e1003965. doi: 10.1371/journal.pmed.1003965 (PMC9075662; doi:10.1371/journal.pmed.1003965)
Supplement: S2 Fig — (DOCX) [file pmed.1003965.s004.docx]

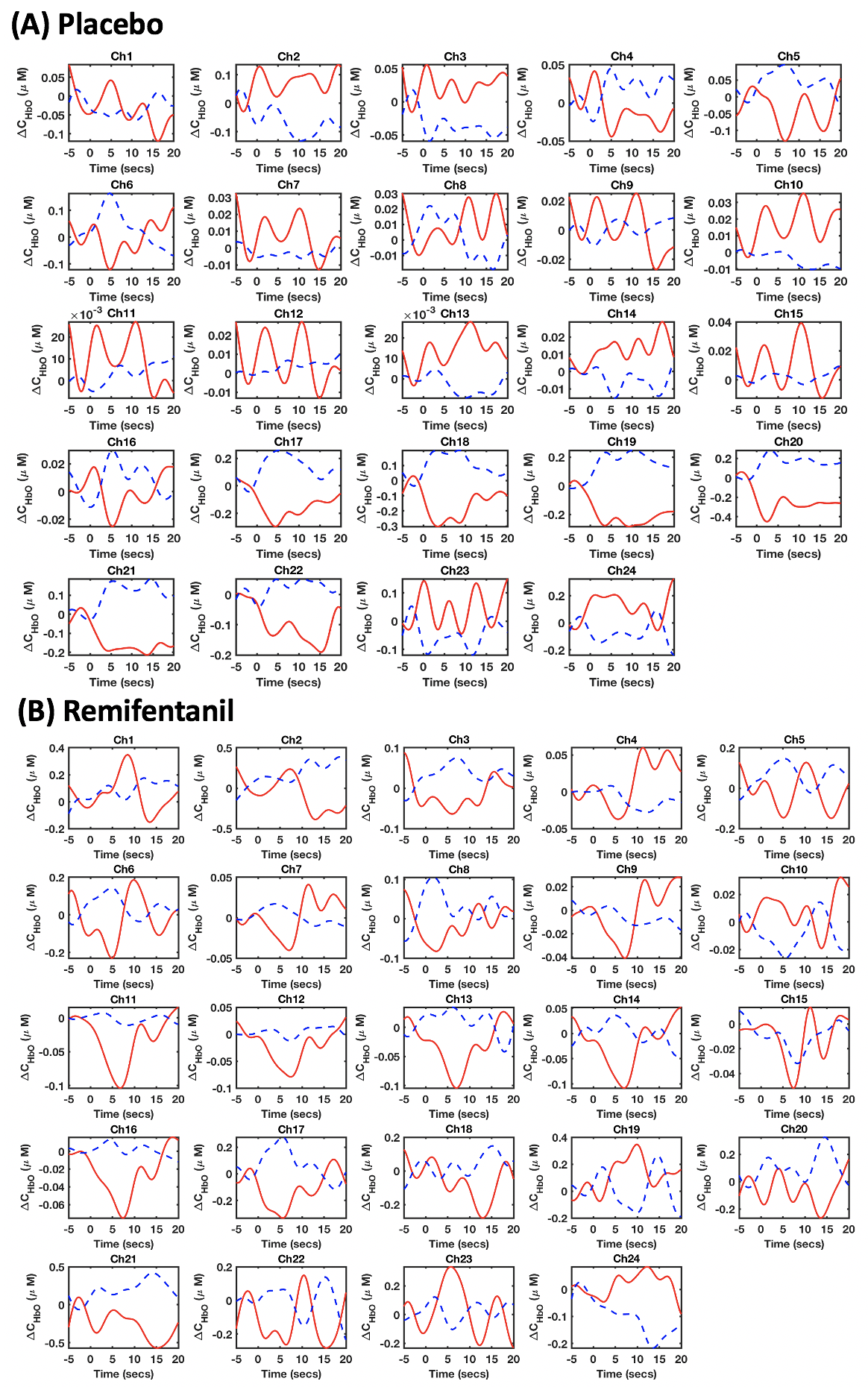


**S2 Fig:** **Group-Averaged Hemodynamic Response to Ablation in all 24 Channels:** Average hemodynamic response to ablation in (A) Placebo and (B) Remifentanil groups for all 24 channels. Red represent changes in oxy-hemoglobin concentration and blue dotted lines indicated deoxy-hemoglobin concentration change.
