# Supplementary material for: Brain-based measures of nociception during general anesthesia with remifentanil: A randomized controlled trial
Source: PLoS Med. 2022 Apr 22;19(4):e1003965. doi: 10.1371/journal.pmed.1003965 (PMC9075662; doi:10.1371/journal.pmed.1003965)
Supplement: S3 Fig — (DOCX) [file pmed.1003965.s005.docx]

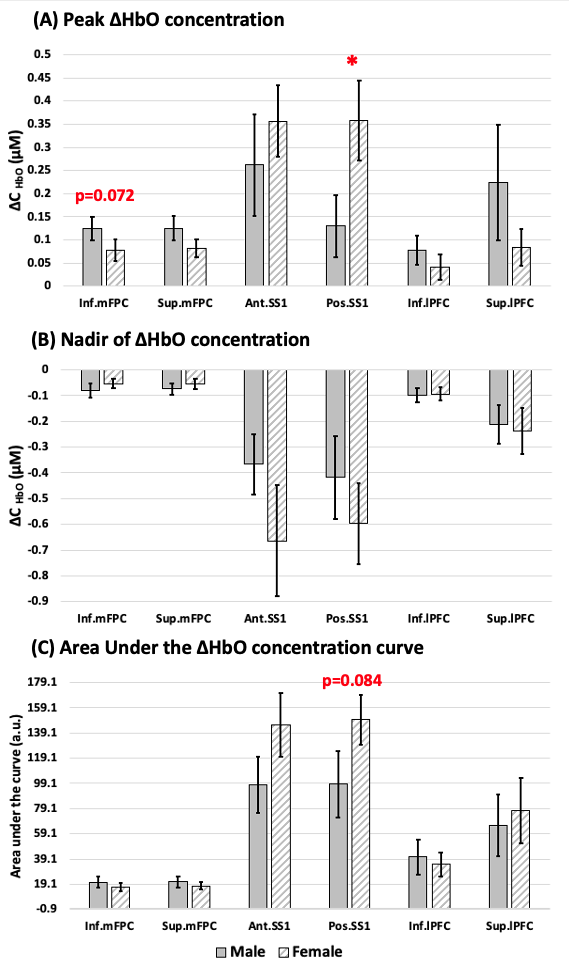


**S3 Fig : Sex-related effects in response to ablation in male vs female patients >14 years of age during general anesthesia:** (A) Peak ΔHbO concentration, (B) Nadir of ΔHbO concentration and (C) Area under the DHbO curve. * indicates significant effects between male and female patients using two-sample t-tests at uncorrected-p<0.05. Error bars represent the standard error of mean. **Abbreviations:** Inf. mFPC, inferior medial frontopolar cortex; Sup. mPFC, superior medial frontopolar cortex; Ant. SS1, anterior superior somatosensory cortex; Pos. SS1, posterior superior somatosensory cortex; Inf. lPFC, inferior lateral prefrontal cortex; and Sup. lPFC, superior lateral prefrontal cortex.
