# Supplementary material for: Brain-based measures of nociception during general anesthesia with remifentanil: A randomized controlled trial
Source: PLoS Med. 2022 Apr 22;19(4):e1003965. doi: 10.1371/journal.pmed.1003965 (PMC9075662; doi:10.1371/journal.pmed.1003965)
Supplement: S4 Fig — AUC, area under the ΔHbO curve. (DOCX) [file pmed.1003965.s006.docx]

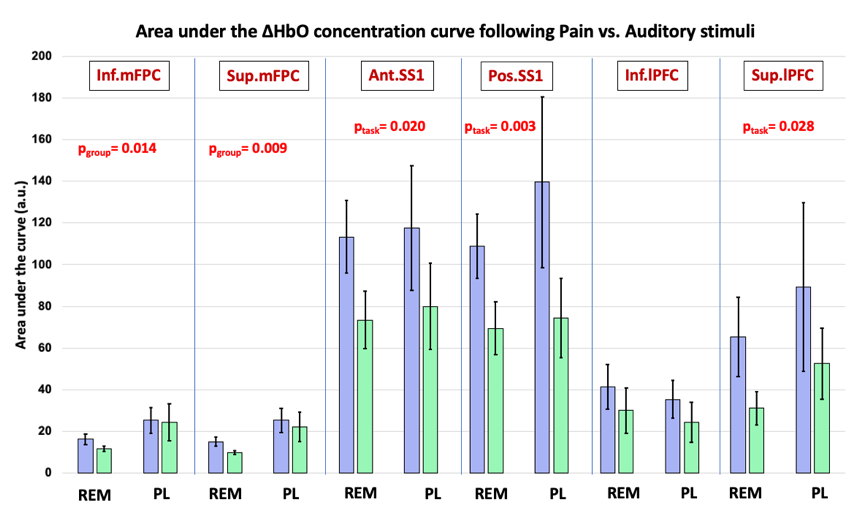


**S4** **Fig: AUC measures following Painful vs Auditory Stimuli under General Anesthesia:** AUC of ΔHbO concentration following painful vs auditory stimuli in the six regions of interest classified based on drug group. Mixed ANOVA results at uncorrected p<0.05 where p_task_ indicates the pvalue for mean effect of task (Pain vs Audio); p_group_ indicates the p-value for mean effect of drug group (Placebo vs Remifentanil); p_group x task_ indicates the p-value for interaction effect of task and drug group (Pain vs. Audio in Placebo and Remifentanil). **Abbreviations:** Inf. mFPC, inferior medial frontopolar cortex; Sup. mPFC, superior medial frontopolar cortex; Ant. SS1, anterior superior somatosensory cortex; Pos. SS1, posterior superior somatosensory cortex; Inf. lPFC, inferior lateral prefrontal cortex; and Sup. lPFC, superior lateral prefrontal cortex.
