# Supplementary material for: Brain-based measures of nociception during general anesthesia with remifentanil: A randomized controlled trial
Source: PLoS Med. 2022 Apr 22;19(4):e1003965. doi: 10.1371/journal.pmed.1003965 (PMC9075662; doi:10.1371/journal.pmed.1003965)
Supplement: S1 Table — (DOCX) [file pmed.1003965.s007.docx]

S1 Table Results of independent sample t-test comparing cortical activation measures during ablation between male and female patients

|  | | **Peak ΔHbO** | | **Nadir of ΔHbO** | | **Area under the ΔHbO curve** | |
| --- | --- | --- | --- | --- | --- | --- | --- |
| **Region** | | **Mean ± SEM** | **p-value** | **Mean ± SEM** | **p-value** | **Mean ± SEM** | **p-value** |
| **Inf.mFPC** | M | 0.117 ± 0.021 | 0.250 | -0.080 ± 0.023 | 0.532 | 19.954 ± 3.495 | 0.573 |
|  | F | 0.080 ± 0.022 |  | -0.058 ± 0.017 |  | 16.781 ± 3.168 |  |
| **Sup.mFPC** | M | 0.117 ± 0.022 | 0.220 | -0.076 ± 0.018 | 0.453 | 19.372 ± 3.499 | 0.757 |
|  | F | 0.079 ± 0.018 |  | -0.054 ± 0.019 |  | 17.462 ± 2.678 |  |
| **Ant.SS1** | M | 0.259 ± 0.089 | 0.406 | -0.326 ± 0.099 | 0.216 | 91.911 ± 18.495 | 0.110 |
|  | F | 0.348 ± 0.072 |  | -0.630 ± 0.205 |  | 138.993 ± 24.236 |  |
| **Pos.SS1** | M | 0.127 ± 0.054 | 0.012 | -0.363± 0.133 | 0.295 | 90.531 ± 22.006 | 0.057 |
|  | F | 0.351 ± 0.080 |  | -0.567 ± 0.150 |  | 143.424 ± 19.688 |  |
| **Inf.lPFC** | M | 0.075 ± 0.026 | 0.424 | -0.101 ± 0.025 | 0.762 | 39.687 ± 11.499 | 0.695 |
|  | F | 0.042 ± 0.026 |  | -0.089 ± 0.025 |  | 33.930 ± 8.992 |  |
| **Sup.lPFC** | M | 0.185 ± 0.102 | 0.387 | -0.183 ± 0.062 | 0.667 | 57.447 ± 20.399 | 0.586 |
|  | F | 0.080 ± 0.037 |  | -0.226 ± 0.084 |  | 73.662 ± 24.384 |  |

‘M’ denotes males and ‘F’ denotes females. SEM is the standard error of mean.
