# Supplementary material for: Brain-based measures of nociception during general anesthesia with remifentanil: A randomized controlled trial
Source: PLoS Med. 2022 Apr 22;19(4):e1003965. doi: 10.1371/journal.pmed.1003965 (PMC9075662; doi:10.1371/journal.pmed.1003965)
Supplement: S1 Protocol — (PDF) [file pmed.1003965.s008.pdf]

Title: Assessing the Cortical Response to Noxious and Auditory Stimuli Using Near Infrared Spectroscopy in Subjects under General Anesthesia

Research Question: To determine the utility of functional near infrared spectroscopy (fNIRS) in the objective, quantitative assessment of nociception and anti-nociception (analgesia) in patients under general anesthesia. A secondary question is the use of fNIRS to measure actual or intended movement either spontaneously, in response to pain, and/or in response to auditory stimuli during general anesthesia.

Significance: This study is part of an ongoing project to determine the value of fNIRS/DOI as a tool for measurement of nociception and anti-nociception and monitoring depth of anesthesia to prevent awareness and pain in patients under general anesthesia. NIRS/DOI is capable of continuously monitoring and tomographically imaging changes in oxy- and deoxyhemoglobin concentrations as a result of noxious stimulation.

Design: Prospective cohort study.

Subjects: Pediatric and adult subjects 12-30 years of age (ASA physical status I or II) undergoing routine electrophysiology procedures in the cardiac catheterization laboratory at Boston Children's Hospital will be eligible for enrollment. We plan to study and obtain useful data sets in 45 subjects with structurally normal hearts undergoing catheter ablation of arrhythmias under planned general endotracheal anesthesia.
Parents/guardians of eligible children and adults will be approached for enrollment at the pre-catheterization evaluation

Variables: Changes in oxyhemoglobin and deoxyhemoglobin concentrations measured by NIRS. Functional NIRS, also known as diffuse optical imaging (DOI), will be used to measure (1) the hemodynamic activations in the bilateral somatosensory cortices and the prefrontal cortices in response to the noxious stimulation (pain) associated with catheter ablation of arrhythmias, and whether these activations are reduced or eliminated by remifentanil, and (2) the hemodynamic activations in the supplemental motor area to actual or intended movement either spontaneously, in response to pain, and/or in response to auditory stimuli.

Statistical Issues: In our pilot study, we were able to collect useable data from five of the eleven subjects that were enrolled (45%). In the five subjects that produced usable results, the signal of interest had a mean value of -3.764×10^{-7} Moles with a standard deviation of 2.135×10^{-7} Moles. This results in a standardized effect size of 1.763, when using a zero mean for the null case. In order to achieve a 90% power level for this measure, the number of usable data sets is therefore eight. Considering our previous success rate of 45%, approximately 18 subjects will need to be enrolled into each group of the study for a total enrollment of 54 subjects. The data analysis will first be performed on a within-subject basis. Then, the original raw optical time series will be averaged across subjects (for corresponding optode placements only, standardized relative to the EEG 10-20 system).

1. Specific Aims /Objectives

The primary aim is to utilize near-infrared spectroscopy (NIRS) in patients under general anesthesia to measure hemodynamic activations in the bilateral somatosensory cortices and the prefrontal cortices in response to the noxious stimulation (pain) associated with catheter ablation of arrhythmias. Randomization of patients to placebo or one of two opioid doses should allow for discrimination between cortical activation and scalp sympathetic changes in response to pain.

Secondary aims are to utilize NIRS to measure hemodynamic activation in the supplemental motor area to actual or intended movement either spontaneously or in response to pain, and in the auditory cortices in response to auditory stimuli.

This study is part of an ongoing project to determine the value of NIRS as a tool for measurement of pain during general anesthesia and to monitor depth of anesthesia in order to prevent awareness.

2. Background and Significance

Anesthesia is a state of drug-induced unconsciousness with suppression of sensory perception, differing from normal sleep by the intensity of stimulus required to awaken the brain to conscious perception. Anesthesia can be defined by hypnotic (unconsciousness) and analgesic (pain relief) components, so that clinical anesthesia involves the administration of hypnotic and analgesic agents with the anesthesiologist observing the clinical response to noxious stimuli and adjusting the dosage to achieve an adequate depth of anesthesia.

There is accumulating evidence that inadequate analgesia has measurable effects on brain structure and function. Pain or inadequate analgesia can alter the neurological development and plasticity of the developing brain (Lowery, Hardman et al. 2007), and surgical trauma can result in significant chronic pain (Kehlet, Jensen et al. 2006, Bingel, Schoell et al. 2007).

Awareness during general anesthesia ('intraoperative awareness') involves the patient having some recollection of events during his or her surgery, including the inability to communicate, helplessness, terror, feeling sensations, experiencing pain, and/or hearing sounds. Awareness can be associated with significant emotional distress and post-traumatic stress disorder (PTSD) (Osterman, Hopper et al. 2001, Lennmarken and Sydsjo 2007, Samuelsson, Brudin et al. 2007). The incidence of intraoperative awareness with recall varies across patient populations and institutions, but is estimated to range from 0.1% to 0.21% in adults (Sandin, Enlund et al. 2000, Sebel, Bowdle et al. 2004, Avidan, Zhang et al. 2008), with a higher (0.8%) incidence in children (Davidson, Huang et al. 2005, Malviya, Galinkin et al. 2009). Intraoperative awareness is likely to be significantly underreported, as patients may not disclose an awareness experience spontaneously (Samuelsson, Brudin et al. 2007) and may not recall intraoperative events (Kerssens, Gaither et al. 2009).

The adequacy of anesthesia is monitored principally by observation of the clinical response to noxious stimuli as a measure of analgesia and the electroencephalogram (EEG) as a measure of hypnosis. Improvements in signal processing have resulted in several commercial monitors that use processed EEG parameters and proprietary algorithms to measure depth of anesthesia. The

most extensively researched system is the Bispectral Index (BIS, Aspect Medical Systems, Norwood, MA), with current evidence indicating that BIS monitoring may reduce, but not eliminate, intraoperative awareness (Ekman, Lindholm et al. 2004, Myles, Leslie et al. 2004, Avidan, Zhang et al. 2008). There is no objective measure of awareness or pain in the operating room. This is especially an issue in the pediatric population that is pre-verbal, or developmentally delayed, where communication after the fact is still not possible. Although numerous articles on the use of BIS have been published, and the application of the system has been used in millions of patients undergoing surgical anesthesia, the system does not measure specific regional brain function such as awareness or pain. Without more sensitive objective measures such conditions may continue to go on "unaware" by clinicians. Part of the interaction between awareness and pain is that patients sometimes have lighter anesthesia for safety reasons (e.g., in obstetrics to limit anesthetic effects on the fetus, in trauma cases and patients with cardiac disease where physiological stability is compromised).

It has been shown by functional magnetic resonance imaging (fMRI) that subjects in a vegetative state (awake but show no clinical signs of awareness) may still experience awareness (Owen, Coleman et al. 2006, Monti, Vanhaudenhuyse et al. 2010). Such a notion has significant implications in anesthesia for preventing unintended awareness or subconscious processing of pain. As fMRI is not practical for use as a routine monitor, and considering the complexity of brain function, there is a pressing need for development of a monitor for intraoperative assessment of awareness and pain.

Near infrared spectroscopy (NIRS) is a spectroscopic method that uses light in the near-infrared region of the electromagnetic spectrum. Near-infrared light (700-1000 nm) penetrates superficial layers (skin, subcutaneous fat, skull, etc.) and is either absorbed by chromophores (oxy- and deoxyhemoglobin) or scattered within the tissue. NIRS is a noninvasive and relatively low-cost optical technique that is becoming a widely used instrument for measuring tissue oxygen saturation, changes in hemoglobin volume and, indirectly, brain blood flow and oxygen consumption (Ferrari, Mottola et al. 2004). NIRS can also be used for non-invasive assessment of brain function through the intact skull by detecting changes in blood hemoglobin concentrations associated with neural activity, and may be considered as a partial replacement for fMRI. It can be used on infants and children, allowing for freely moving subjects since the monitors are applied to the head. Because of limitations of light penetrance, NIRS can only be used to measure brain activity in cortical regions. NIRS has been used to measure the cortical activation in response to tactile, thermal, and painful stimuli (Becerra, Harris et al. 2008, Becerra, Harris et al. 2009, Yucel, Aasted et al. 2015).

The optical imaging system we plan to use for this project is the Continuous Wave (CW) 7 Near-Infrared Spectrometer (TechEn Inc., Milford, MA). The TechEn CW 7 is intended for the continuous noninvasive monitoring of changes in hemoglobin during functional activities (such as touch, pain, thermal sensation, movement). The TechEn CW 7 is principally the same as the TechEn CW 6 but with new features that include smart lasers with software intensity power control, short distance probes, and other techniques to improve ease of use and signal quality (Appendix A). The TechEn CW 6 is currently used only for research applications and includes monitoring tissue oxygenation in brain, breast, and muscle. The TechEn CW 6 device has been approved for clinical research in other studies at Boston Children's Hospital (PI: Ellen Grant MD

MSc, Protocol Number 2003P001728) and other Harvard institutions including the Massachusetts General Hospital (IRB #'S 2007-P-000270; 2007-P-001614; 2009-P-001615; 2009-P-002821). The system has 32 diode lasers (emitting at 690 and 830 nm) and 32 detectors. The sources and detectors are coupled to fiber optics, with each emitting fiber and detector constituting one optode; the optodes are 1-3mm in diameter, allowing them to be easily wiggled through the hair to make optical contact with the scalp. The optical probe comprises the 32 optodes (see photo below). TechEn provides excellent support and training.

Our current publications and preliminary data (Figures 1 and 2) show that fNIRS can be used to measure the specific cortical activity associated with the sensation of pain and can distinguish noxious and innocuous stimuli (Yucel, Aasted et al. 2015).

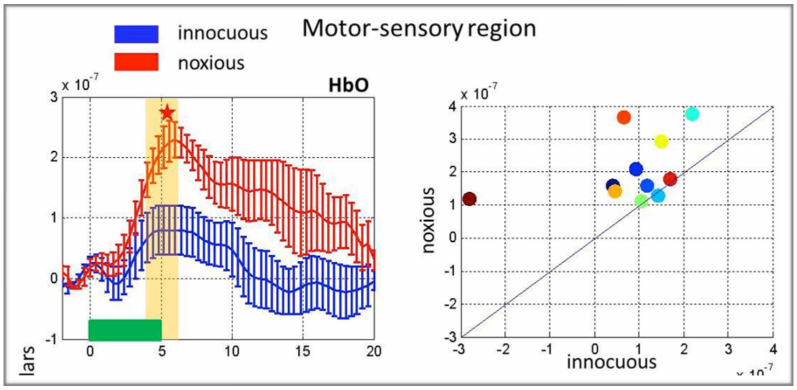

Figure 1: Characterization of Pain Response: Changes in HbO (top, left) and HbR (bottom, left) as a response to innocuous stimuli (blue) and noxious stimuli (red). Yellow bars show the interval chosen to obtain the mean responses depicted in the scatter plots and stars indicate a statistically significant difference. The right panels show a scatter plot comparing the hemodynamic response for each subject averaged over the yellow bar during the first three minutes for HbO (top) and HbR (bottom). The horizontal green bar shows the stimulus duration (Yucel, Aasted et al. 2015).

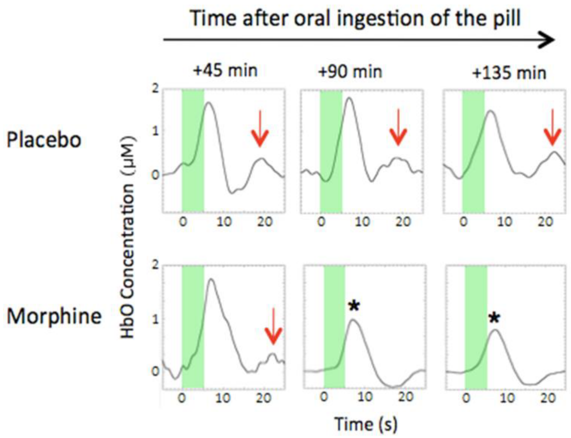

Figure 2: Effects of Oral Morphine on the fNIRS HbO pain signal. The magnitude (*) of the response is clearly reduced by analgesia and the second peak is eliminated (red arrows) (n=1).

These results strongly suggest that fNIRS has the potential to provide a biomarker for intraoperative pain.

The primary objective of this proposal is to determine the utility of an fNIRS approach in the objective, quantitative assessment of analgesia in a surgical environment. In achieving this goal we would provide anesthesiologists with the ability to better manage patient analgesia and would ultimately reduce the incidence of post-operative sequelae, including chronic neuropathic pain. An objective measure of pain signaling in anesthetized patients would not only promote appropriate analgesia during surgery but would allow the impact of analgesia on postoperative recovery and long-term outcomes to be quantified. By using measures that focus on the specific cortical responses associated with the sensation of pain and the activation of brain networks associated with pain, the successful outcome of this study will give rise to clinical trials that could fundamentally improve how pain is managed during and after surgery, leading to improved patient outcomes and reduced health care costs.

3. Preliminary Studies/Progress Report

We performed a pilot study at BCH (Protocol #10-07-0336) to investigate the feasibility of NIRS to measure cortical signals produced by noxious stimulation in patients under general anesthesia. In this study, we found that catheter ablation (radiofrequency or cryoablation) of arrhythmias produced cortical signals that mirror those seen with noxious stimulation in healthy volunteers and fMRI activations in response to pain. This work is being prepared for submission to a peer-reviewed journal.

Our preliminary results with a continuous-wave (CW) imaging system in healthy adults (work performed at the Massachusetts General Hospital) support our hypothesis that it is possible to

measure pain stimuli in the hand sensory area, and to co-register the optical signal with fMRI measurements. We have demonstrated that NIRS, also known as diffuse optical imaging (DOI), is capable of continuously monitoring and tomographically imaging changes in oxy-and deoxyhemoglobin concentrations during somatosensory stimulation, such as finger tapping, finger tactile, and electrical median nerve stimulation (Becerra, Harris et al. 2008, Minati, Jones et al. 2009, Nambu, Osu et al. 2009) and multiple applications in children (Wolf and Greisen 2009, Watanabe, Homae et al. 2010). We have also shown with fMRI studies that pain stimulation activates sensory areas. Our group has a great deal of experience performing DOI measurements simultaneously with fMRI, and have demonstrated that the functional signals measured with DOI correlate temporally with fMRI signals (Strangman, Culver et al. 2002).

In our previous work using the same approach we have shown that an acute thermal stimulus using an FDA-approved Peltier thermode produces significant and measurable effects that can be measured in a number of cortical regions in healthy volunteers (Becerra, Harris et al. 2008). We have not completed the work on simultaneous fMRI and DOT measures in healthy subjects and this continues under an approved IRB protocol at MGH, 2007-P-000270, entitled "Assessing the Cortical Response to Painful Stimuli with Diffuse Optical Imaging".

4. Design and Methods

a. Study Design

General Procedures

We plan to study and obtain useful data sets in 45 subjects (12-30 years of age) scheduled for elective electrophysiology procedures in the cardiac catheterization laboratory and undergoing ablation of arrhythmias under planned general endotracheal anesthesia. As a number of subjects may choose to withdraw from the study following enrollment, and because some subjects will not be able to participate in the study if it is not possible to obtain adequate NIRS signals (as discussed below under Study Procedure: Day of Surgery), we plan to enroll 54 subjects to allow for drop-outs.

- Pre-Operative Visit

The pre-operative visit for electrophysiology procedures typically takes place in the cardiac pre-operative clinic 1-2 days before the date of catheterization. For patients currently in hospital, evaluation and recruitment will take place on the ward, typically the day prior to the procedure. At this visit, the Principal Investigator, an Anesthesia Research Nurse, and/or an Anesthesia Research Coordinator will present the study to the subject or to the parent/guardian and child (if the subject is a minor). At this time, consent and child assent when appropriate will be obtained. Following consent/assent, a Pre-Operative Questionnaire (Appendix B) will be filled out by the subject. This questionnaire asks study-relevant questions, such as hair color and thickness, and questions regarding demographics and subject contact information. If preferred, the subject may take the questionnaire home to fill out and return it to the study staff prior to the procedure.

After consent has been obtained, the subject will be assigned a study identification number and randomized to receive during the procedure normal saline infusion (placebo; i.e. no remifentanil) or to one of two remifentanil infusion regimens. The study subject and investigators analyzing the NIRS signals will be blinded to this information.

- Day of Surgery

<u>Before the Procedure</u>:

First, the subject will be rebriefed on the nature of the study and of the experimental paradigms. Subjects will have the option of withdrawing from the study prior to administration of sedation and anesthesia.

Following light sedation with midazolam and prior to induction of general anesthesia, study personnel will position the probe holding the fiber optics ("optodes") on the subject's head. The optodes are contained in a cap (shown below). The cap is not constricting, and any discomfort will be minimized by surrounding the optodes with foam tape and using soft cloth straps against the head, face and chin. After initial positioning, the cap may need to be shifted slightly until adequate signals are obtained through all detector fibers. The fibers are 1-3mm in diameter, allowing them to be easily wiggled through the hair to make optical contact with the scalp. In some cases, hair will be parted in order to achieve better optical contact. No shaving of hair will be necessary or performed.

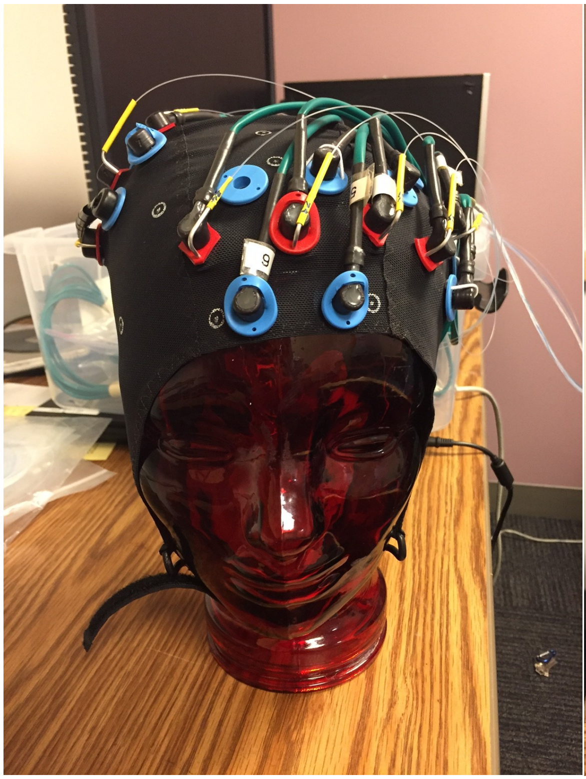
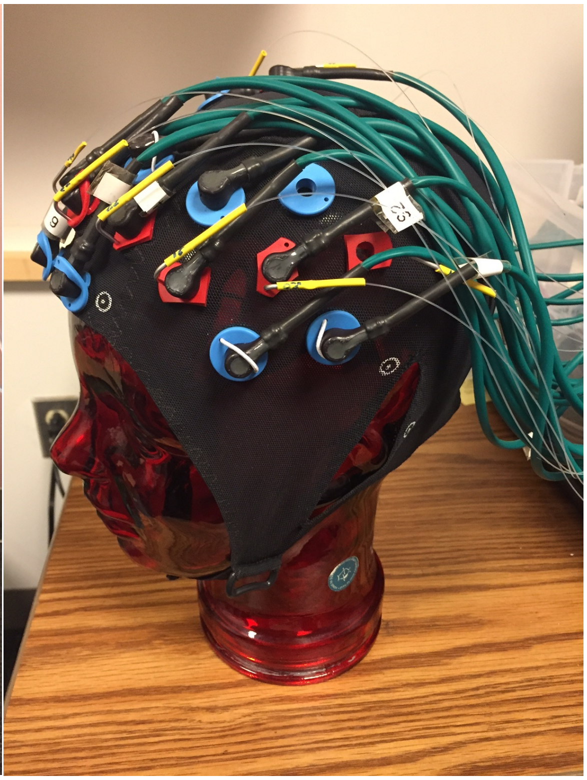

Prior to the induction of general anesthesia, we will perform the following to test optode placement and signal quality:

1. Resting state data will be collected for about 200 seconds while the patient is quietly lying still. This baseline data will be used to compare changes observed during the catheterization procedure.

2. Auditory Stimuli: Headphones will be placed on the subject's head and an audio recording will be presented to the subjects. This data will be used as baseline consciousness data, as well as to familiarize the subject with the study procedures. In order to activate the Supplemental Motor Area (SMA), which should be quiet unless patients move, we will adapt a previously employed method for evaluating subjects in the vegetative state (Owen, Coleman et al. 2006). The auditory stimulus will be presented for 5 seconds, with a varied inter-stimulus interval. The prompt will request that the subject imagine squeezing a tennis ball in their right hand in a clasping motion, without actually performing the action. During this we expect SMA activity to occur if the subject is consciously aware.

3. . The subject will be asked to perform a brief finger tapping exercise, tapping the fingers of the left hand against the thumb for 5 seconds, with 15 seconds between, for 5 cycles. This will only be conducted if time permits.

4. . Brush stimuli will be applied to the dorsum of the left hand in a similar manner. This will only be conducted if time permits.

4.

A BIS (Bispectral Index) monitor will be used in all study patients in order to control the depth of anesthesia. A BIS sensor will be placed on the forehead and a BIS value between 40 and 60 will be targeted. A BIS <60 is advocated to prevent awareness during general anesthesia (Avidan, Zhang et al. 2008). This will also allow for reduced variability in the depth of anesthesia between the subjects during the ablations.

<u>During the Procedure</u>:

The audio stimuli will be presented to the subject every half hour throughout the procedure. Near-infrared signals are measured and recorded by the NIRS device (TechEn CW 7) throughout the procedure and will not influence the procedure.

The anesthetic technique will be standardized for all study subjects, and will comprise the following:

Premedication: Midazolam 0.05 mg/kg IV up to a maximum of 2 mg

Induction: Fentanyl 1.5 mcg/kg IV up to a maximum of 3 mcg/kg. No fentanyl after induction.
Propofol – standard dosage titrated to effect

Rocuronium – standard dosage

Maintenance: Randomization to 3 groups, with infusion begun after induction while the patient is being prepped. Infusions to run until cardiologist informs anesthesiologist that no further ablations will be performed. Infusions will be prepared by the hospital pharmacy.

- Group 1: Placebo (0.9% NaCL)
- Group 2: Remifentanil 0.25 mcg/kg/min
- Group 3: Remifentanil 0.5 mcg/kg/min

Sevoflurane end-tidal concentration 1 to 4% (equivalent to 0.5 to 2 MAC)
Rocuronium – standard dosage
Ondansetron 0.1 mg/kg following last ablation

Emergence: Prior to emergence from anesthesia, the catheter access sites will be infiltrated with lidocaine as is the usual practice, the catheters removed, muscle relaxation reversed, and the patient allowed to awaken normally from anesthesia. The headphones and probe cap will be removed from the subject's head prior to emergence from anesthesia. The BIS sensor will be removed just before or just after emergence from anesthesia.

The only substantive difference between the study protocol and standard clinical care is the administration of remifentanil. Intravenous opioids are typically not given beyond induction of general anesthesia nor during catheter ablation of the arrhythmia. In this study, there is no reduced pain control than normal.

After the Procedure:

In the recovery room, before the subject is discharged from the hospital, a Post-Operative Questionnaire (Appendix C) will be performed in which the subject is asked about the last thing they remember before going to sleep, the first thing they remember upon waking up, and whether they have any memories or were dreaming during the procedure. If the research team is unable to administer the Post-Operative Questionnaire prior to the subject being discharged from the hospital, the subject will be contacted by telephone to complete this.

Additionally, information pertaining to the study will be collected from the electronic medical record. This information will include data from the pre-operative evaluation, electrophysiology procedure, anesthesia administered, and the recovery period.

b. Patient Selection and Inclusion/Exclusion Criteria

Inclusion Criteria:

12-30 years of age
Scheduled for electrophysiology procedure (EP) under general endotracheal anesthesia
Structurally normal heart by echocardiography
Right-handed

Exclusion Criteria:

- Structural heart disease
- Left handed
- Unable to cooperate or understand the nature of the study
- Significant medical history, including neurologic or muscular disease, diabetes mellitus, syndromes of greater than minor severity.
- Smoking
- Subject's scalp or hair does not permit sufficient optical light detection
- Subject unable to keep his/her head still for a period of 200 consecutive seconds (so as to obtain adequate baseline signals).

c. Recruitment Methods

i. HOW, WHERE and WHEN will potential subjects be recruited?

Fifty-four subjects will be recruited when scheduled for an elective electrophysiology procedure in the cardiac catheterization laboratory and with a high likelihood of catheter ablation of an arrhythmia. Patient identification will occur by checking the scheduled electrophysiology cases and reviewing the patient's medical record for the appropriate inclusion and exclusion criteria. Identification will be performed by the Anesthesia Research Nurse or Anesthesia Research Coordinator, after which the PI will review the medical record to ensure suitability. Potential subjects will be approached and recruited by one of these individuals at the scheduled pre-catheterization evaluation. The evaluation typically takes place in the cardiac pre-operative clinic 1-2 days before the date of catheterization. For patients currently in hospital, identification, evaluation and recruitment will take place on the ward, typically the day prior to the procedure.

ii.WHAT recruitment methods and materials (e.g. posters, fliers) will be used?

Recruitment will be done orally by the Anesthesia Research Nurse, Anesthesia Research Coordinator, or Principal Investigator using the Research Consent Form to present the study. Informed consent will be obtained in the cardiac pre-operative clinic; for those patients currently in hospital informed consent will be obtained on the ward.

iii.WHO will be responsible for subject recruitment?

The Anesthesia Research Nurse, Anesthesia Research Coordinator, and/or Principal Investigator (Barry Kussman, MBBCh).

d. Description of Study Treatments or Exposures/Predictors

The study involves two aspects:

1. The near-infrared imaging technique utilizes the Continuous Wave Near Infrared Spectrometer, (TechEn, Inc., Milford, MA). The system has 32 diode lasers (emitting at 690 and 830 nm) and 32 detectors. The sources and detectors are coupled to fiber

optics, with each emitting fiber and detector constituting one optode; the optodes are 1-3mm in diameter, allowing them to be easily wiggled through the hair to make optical contact with the scalp. The optodes are contained within a cap which will be placed on the patient's head in the catheterization laboratory. The light passes harmlessly through the subject's scalp onto the brain surface and returns, with some attenuation, to the detector optodes. The returning light yields information about blood-oxygenation levels in that region of the brain. The probe cap is not constricting and any discomfort will be minimized by surrounding the optodes with foam tape and using soft cloth straps against the head and face. After initial positioning, the probe may need to be shifted slightly until adequate signal is obtained through all detector fibers. In some cases, hair will be parted in order to achieve better optical contact. There will be no shaving of hair.

Prior to induction of general anesthesia, baseline activation signals of the somatosensory cortices and prefrontal cortices will be obtained in response to finger-tapping and brush stimuli applied to the dorsum of the hand as described above. This will provide baseline information to compare to the signals obtained during the procedure from surgically-induced pain.

In order to activate the Supplemental Motor Area, which should be quiet unless patients move, headphones will be placed on the subjects and an audio recording will be presented to subjects. The prompt will request that the subject imagine squeezing a tennis ball in their right hand, in a clasping motion, without actually performing the action. During this we expect SMA activity to occur if the subject is consciously aware based on similar results from prior studies. The audio stimuli will be presented to the subject every half hour throughout the procedure.

2. Patients will be randomized into one of three groups to receive an infusion which will be started after induction of general anesthesia while the patient is being prepped.
   Group 1: 0.9% NaCl infusion
   Group 2: Remifentanil 0.25 mcg/kg/min infusion
   Group 3: Remifentanil 0.5 mcg/kg/min infusion

   The infusions will be prepared by the hospital pharmacy. Investigators performing analysis of the NIRS signals will be blinded to the infusion the patient received. Infusions will be stopped after the cardiologist informs the anesthesiologist that no further ablations will be performed.

The only substantive difference between the study protocol and standard clinical care is the administration of remifentanil. Intravenous opioids are typically not given beyond induction of general anesthesia nor during catheter ablation of the arrhythmia.

e. Definition of Primary and Secondary Outcomes/Endpoints

Primary Outcomes:

The primary outcome is the use of NIRS to measure/detect hemodynamic activations in the bilateral somatosensory cortices and the prefrontal cortices in response to the noxious stimulation (pain) associated with catheter ablation of arrhythmias, and whether these activations are reduced or eliminated by remifentanil.

Secondary Outcome:

The secondary outcome is the use of NIRS to measure hemodynamic activation in the supplemental motor area to actual or intended movement either spontaneously, in response to pain, and/or in response to auditory stimuli.

f. Data Collection Methods, Assessments and Schedule (what assessments performed, how often)

The study will consist of enrollment and a Preoperative Questionnaire at the pre-catheterization work-up, the study protocol during two days if the patient is discharged the same day of the procedure, or three days if the subject is admitted overnight.

Prior to Preoperative Visit:

- Assessment of eligibility criteria

Pre-Operative Visit:

- Presentation of research study, informed consent/assent (20 minutes)
- Preoperative Questionnaire if patient/parents consent/assent (10 minutes)

Day of Procedure:

1) Before Procedure
- Equipment setup (20 minutes). Test run of brush and audio stimuli (10 minutes).
- BIS sensor applied to patient.

2) During Procedure
- Infusion of NaCL or remifentanil started after induction of anesthesia.
- Audio stimuli (5 seconds each) presented to the patient every half an hour.
- Electronic anesthesia record will capture relevant anesthetic and physiologic data. Study Coordinator and/or Research Nurse will document time and duration of ablations, times of catheter insertion and removal, and assure safety of the patient
- An investigator will operate the NIRS equipment.
- NIRS cap with optodes will be removed from the patient just prior to emergence from anesthesia.
- Average duration of an electrophysiologic study with catheter ablation of arrhythmias is 3 to 4 hours.

3) After Procedure
- Postoperative Questionnaire (10 minutes) will be administered before subject is discharged from the hospital, either on the day of or day following the procedure.

- Postoperative Questionnaire (10 minutes) will be readministered one month following the procedure.

g. Study Timeline (as applicable)

The subject's total time commitment, as detailed above, will be approximately 5 to 6 hours, including the duration of the electrophysiologic procedure. The equipment set up will be done prior to the induction of anesthesia, resulting in no increase in the duration of anesthesia.

In addition, information pertaining to the study will be collected from the electronic medical record. This information will include data from the pre-operative evaluation, electrophysiology procedure, anesthesia administered, and the recovery period.

h. Adverse Event Criteria and Reporting Procedures
Adverse events related to the study will be brought to the immediate attention of the Principal Investigator and the patient's medical and nursing staff. Adverse events would include skin irritation or injury from the optodes and cap straps. Adverse events will be reported by the study investigators to the IRB. Peri-catheterization complications not related to study interventions will not be reported.

5. Data Management and Statistical Analysis

a. Data Management Methods

Boston Children's Hospital is the sole site for enrollment and performance of the study. Research folders will be stored in the Anesthesia Research office in a locked cabinet. NIRS data collected will be stored and analyzed by the P.A.I.N. Group members on this study, either on secure electronic servers or in secure office spaces. Data will be de-identified; any data that is linked to the subject's identity will be kept in a locked cabinet in the Anesthesia Research office and P.A.I.N. group office space.

b. Quality Control Method

Training of all research team members who will operate the TechEn CW7 will be mandatory to ensure the accuracy and reliability of the probe placement and signals acquired. See "Data Analysis Plan" for more details on quantitative analysis.

c. Data Analysis Plan

The NIRS instrument will acquire raw data from each detector at ~40 kHz. The individual signals will then be bandpass filtered. Next, the time series pairs from each detector will be converted from wavelength (absorption) to relative concentrations (of oxy-/deoxy-hemoglobin). This will be done with the modified Beer-Lambert Law and the absorption coefficients of oxy- and deoxy-hemoglobin (HbO and Hb, respectively). (The near-IR wavelengths are chosen because oxy-

/deoxy-Hb are the predominant absorbers in the tissue at these wavelengths.) The result will be an oxy-Hb and deoxy-Hb time series associated with each source-detector pair.

For each stimulation run, each smoothed oxy-/deoxy-hemoglobin time series will be examined for periodic amplitude fluctuations. Expected fluctuations include heart rate (~1-3 Hz), breathing and/or "v-waves" (~0.1Hz), as well as any fluctuations induced by the experimental paradigm. If the former sources of noise are substantial, an attempt will be made to filter out these fluctuations (in Fourier space). Any analysis of filtered data will be compared to an analysis of the corresponding non-filtered data. The oxy-hemoglobin portion of the experimentally-induced changes are expected to be 4-9 sec out of phase with the paradigm onsets and offsets (as seen in fMRI work as well as in prior optical recordings).

The data analysis will first be performed on a within-subject basis. Then, the original raw optical time series will be averaged across subjects (for corresponding optode placements only, standardized relative to the EEG 10-20 system).

The biological basis for the signals measured by the optical technique is the same as the basis for fMRI and PET scanning. The regions to be sampled in these pilot studies will be several times larger than single voxels in fMRI scanning. Furthermore, experiments with similar optical instruments indicate that these instruments are at least as sensitive as fMRI. With larger sampling regions (up to a factor of 5 or more), and assuming only comparable sensitivity, the optical imaging should require no more subjects than an fMRI study, and perhaps fewer. Furthermore, optical recording can provide 10-100 times the temporal resolution of fMRI. So, since an optical time series would seem to be at least as good as that coming from an individual voxel (or small collections of voxels) from fMRI data, similar group sizes should be adequate for optical experiments (Boas, Dale et al. 2004).

d. Statistical Power and Sample Considerations

In our pilot study, we were able to collect useable data from five of the eleven subjects that were enrolled (45%). In the five subjects that produced usable results, the signal of interest had a mean value of -3.764×10^{-7} Moles with a standard deviation of 2.135×10^{-7} Moles. This results in a standardized effect size of 1.763, when using a zero mean for the null case. In order to achieve a 90% power level for this measure, the number of usable data sets is therefore eight. Considering our previous success rate of 45%, approximately 18 subjects will need to be enrolled into each group of the study for a total enrollment of 54 subjects.

e. Study Organization

The principal investigator for this study is Barry Kussman, MBBCh in the Department of Anesthesiology, Perioperative and Pain Medicine at Boston Children's Hospital**.** The Research Nurse(s), Study Coordinator (Rachel Bernier MPH), and co-investigators (David Borsook MD, and Lino Becerra PhD) are members of the Department of Anesthesiology, Perioperative and Pain Medicine at Boston Children's Hospital. Other co-investigators (Mark Alexander MD) and (David Boas PhD are members of the Department of Cardiology at Boston Children's Hospital and Martinos Center for Biomedical Imaging, Department of Radiology, Massachusetts General

Hospital), respectively. Members of the P.A.I.N. research group will participate in data collection and data analysis.

f. Data and Safety Monitoring Plan

Confidentiality will be ensured by assigning anonymous subject identification codes to each subject. The subject-screening log, which lists the names corresponding to the subject identification codes, will be kept on a locked computer, access to which is limited to only one investigator.

The risk posed to the subject in this study is minimal. Subjects will have the right to discontinue the study at any time prior to induction for any reason. Also, investigators will be in the room ready to stop the experiment in case there is any sort of adverse event. After induction of anesthesia the attending anesthesiologist and/or cardiologist may discontinue the experiment if clinical conditions warrant or in the case of an adverse event. Adverse events will be reported to the IRB as indicated above.

The investigators will review the data after the first 5 subjects and then after every 10 subjects to ensure that adequate data is being obtained.

6. Risks and Discomforts

DOI: DOI is an investigational tool and though no adverse effects have been reported, it is possible that effects not yet reported may occur. The source lasers used to make the measurements are Class IIIb and use very low power – below the ANSI limit for long-term exposure to infrared light (see chart below). The intensity used to monitor cerebral perfusion parameters is considerably less

Transmitters: Laser Sources

Number of sources	Up to 32
Type of Source	Lasers Class III b
Source wavelengths	690 and 830 nm
Optical Output Power per source	12 mW, 8 mW
Output control capability	On/Off, Square-wave modulation
Connector type	Optical SMA

than the amount of light the brain would receive during an outdoor walk on a sunny day, and therefore is considered to be harmless. Thus far, no hazard to patient, staff or third party has been observed. Should any adverse effects be observed during the study, they will be immediately reported to the IRB.

Operating Room Setting: All of the equipment that is brought into the operating room will follow the standard OR procedures regarding clearance by biomedical engineering and disinfection. Also, after anesthesia is given to the subject the physician or physicians involved in the procedure reserve the right to discontinue participation in the study for the subject if they feel it is in the subject's best interest.

Side Effects of Remifentanil: Remifentanil differs from the other strong opioids in its rapid onset/offset for all clinical effects, including respiration (Dahan, Niesters et al. 2013). Remifentanil is rapidly metabolized by enzymes within red blood cells after cessation of an infusion. As the elimination half-life is 5-8 minutes and the context sensitive half-life is 2 minutes, increased postoperative sedation from the remifentanil is unlikely and the risk of postoperative nausea and vomiting is low compared to the nausea and vomiting associated with the procedure itself. As part of standard of care, the anesthetic plan includes the administration of ondansetron to reduce post-operative nausea and vomiting.

7. Potential Benefits

There is the prospect of a direct benefit to the subject from participating in the study. This study will help researchers more fully understand responses to procedure-induced noxious stimulation and auditory stimuli measured with diffuse optical imaging in subjects during general anesthesia. The information may provide a novel approach in the rational design of methods/therapies for providing anti-nociception and preventing intraoperative awareness.

8. Privacy Provisions

As the outcome of the study will not impact clinical decisions, this information will not become part of any subject's medical record. Potential subjects will be approached during a regularly scheduled visit. Relevant information will be asked from the participants or obtained from their medical records by trained study staff, ensuring privacy. All potential subjects and their decision to participate will be kept confidential and within the research study staff.

9. Confidentiality Provisions

Subjects will be assured of the confidentiality of their information as mandated by the HIPAA regulations of 4/14/03. The data generated by their participation in this study will be maintained in the database via subject coding to protect their identity. All subject related information will be kept in secured, locked cabinets in the Anesthesia Research office and at the P.A.I.N. Group offices, and on the secure servers. Access to any research related records will be limited to the study group. Boston Children's Hospital is the sole site for enrollment and performance of the study. Research folders will be stored in the Anesthesia Research office in a locked cabinet. NIRS data collected will be stored and analyzed by the P.A.I.N. Group members on this study, either on secure electronic servers or in secure office spaces. Data will be de-identified; any data that is linked to the subject's identity will be kept in a locked cabinet in the P.A.I.N. group office space and in locked cabinets in the Anesthesia Research office.

10. Appendices

A. TechEn CW 7 System

B. Preoperative Questionnaire

C. Postoperative Questionnaire

11. References

Avidan, M. S., L. Zhang, B. A. Burnside, K. J. Finkel, A. C. Searleman, J. A. Selvidge, L. Saager, M. S. Turner, S. Rao, M. Bottros, C. Hantler, E. Jacobsohn and A. S. Evers (2008). "Anesthesia awareness and the bispectral index." N Engl J Med **358**(11): 1097-1108.

Becerra, L., W. Harris, M. Grant, E. George, D. Boas and D. Borsook (2009). "Diffuse optical tomography activation in the somatosensory cortex: specific activation by painful vs. non-painful thermal stimuli." PLoS One **4**(11): e8016.

Becerra, L., W. Harris, D. Joseph, T. Huppert, D. A. Boas and D. Borsook (2008). "Diffuse optical tomography of pain and tactile stimulation: activation in cortical sensory and emotional systems." Neuroimage **41**(2): 252-259.

Bingel, U., E. Schoell and C. Buchel (2007). "Imaging pain modulation in health and disease." Curr Opin Neurol **20**(4): 424-431.

Boas, D. A., A. M. Dale and M. A. Franceschini (2004). "Diffuse optical imaging of brain activation: approaches to optimizing image sensitivity, resolution, and accuracy." Neuroimage **23 Suppl 1**: S275-288.

Dahan, A., M. Niesters, E. Olofsen, T. Smith and F. Overdyk (2013). Opiods. Clinical anesthesia. P. G. Barash, B. F. Cullen, R. K. Stoelting et al. Philadelphia, PA, Wolters Kluwer Health/Lippincott Williams & Wilkins: 501-522.

Davidson, A. J., G. H. Huang, C. Czarnecki, M. A. Gibson, S. A. Stewart, K. Jamsen and R. Stargatt (2005). "Awareness during anesthesia in children: a prospective cohort study." Anesth Analg **100**(3): 653-661, table of contents.

Ekman, A., M. L. Lindholm, C. Lennmarken and R. Sandin (2004). "Reduction in the incidence of awareness using BIS monitoring." Acta Anaesthesiol Scand **48**(1): 20-26.

Ferrari, M., L. Mottola and V. Quaresima (2004). "Principles, techniques, and limitations of near infrared spectroscopy." Can J Appl Physiol **29**(4): 463-487.

Kehlet, H., T. S. Jensen and C. J. Woolf (2006). "Persistent postsurgical pain: risk factors and prevention." Lancet **367**(9522): 1618-1625.

Kerssens, C., J. R. Gaither and P. S. Sebel (2009). "Preserved memory function during bispectral index-guided anesthesia with sevoflurane for major orthopedic surgery." Anesthesiology **111**(3): 518-524.

Lennmarken, C. and G. Sydsjo (2007). "Psychological consequences of awareness and their treatment." Best Pract Res Clin Anaesthesiol **21**(3): 357-367.

Lowery, C. L., M. P. Hardman, N. Manning, R. W. Hall and K. J. Anand (2007). "Neurodevelopmental changes of fetal pain." Semin Perinatol **31**(5): 275-282.

Malviya, S., J. L. Galinkin, C. F. Bannister, C. Burke, J. Zuk, M. Popenhagen, S. Brown and T. Voepel-Lewis (2009). "The incidence of intraoperative awareness in children: childhood awareness and recall evaluation." Anesth Analg **109**(5): 1421-1427.

Minati, L., C. L. Jones, M. A. Gray, N. Medford, N. A. Harrison and H. D. Critchley (2009). "Emotional modulation of visual cortex activity: a functional near-infrared spectroscopy study." NeuroReport **20**: 1344-1350.

Monti, M. M., A. Vanhaudenhuyse, M. R. Coleman, M. Boly, J. D. Pickard, L. Tshibanda, A. M. Owen and S. Laureys (2010). "Willful modulation of brain activity in disorders of consciousness." N Engl J Med **362**(7): 579-589.

Myles, P. S., K. Leslie, J. McNeil, A. Forbes and M. T. Chan (2004). "Bispectral index monitoring to prevent awareness during anaesthesia: the B-Aware randomised controlled trial." Lancet **363**(9423): 1757-1763.

Nambu, I., R. Osu, M. A. Sato, S. Ando, M. Kawato and E. Naito (2009). "Single-trial reconstruction of finger-pinch forces from human motor-cortical activation measured by near-infrared spectroscopy (NIRS)." Neuroimage **47**(2): 628-637.

Osterman, J. E., J. Hopper, W. J. Heran, T. M. Keane and B. A. van der Kolk (2001). "Awareness under anesthesia and the development of posttraumatic stress disorder." Gen Hosp Psychiatry **23**(4): 198-204.

Owen, A. M., M. R. Coleman, M. Boly, M. H. Davis, S. Laureys and J. D. Pickard (2006). "Detecting awareness in the vegetative state." Science **313**(5792): 1402.

Samuelsson, P., L. Brudin and R. H. Sandin (2007). "Late psychological symptoms after awareness among consecutively included surgical patients." Anesthesiology **106**(1): 26-32.

Sandin, R. H., G. Enlund, P. Samuelsson and C. Lennmarken (2000). "Awareness during anaesthesia: a prospective case study." Lancet **355**(9205): 707-711.

Sebel, P. S., T. A. Bowdle, M. M. Ghoneim, I. J. Rampil, R. E. Padilla, T. J. Gan and K. B. Domino (2004). "The incidence of awareness during anesthesia: a multicenter United States study." Anesth Analg **99**(3): 833-839, table of contents.

Strangman, G., J. P. Culver, J. H. Thompson and D. A. Boas (2002). "A quantitative comparison of simultaneous BOLD fMRI and NIRS recordings during functional brain activation." Neuroimage **17**(2): 719-731.

Watanabe, H., F. Homae and G. Taga (2010). "General to specific development of functional activation in the cerebral cortexes of 2- to 3-month-old infants." Neuroimage **50**(4): 1536-1544.

Wolf, M. and G. Greisen (2009). "Advances in near-infrared spectroscopy to study the brain of the preterm and term neonate." Clin Perinatol **36**(4): 807-834, vi.

Yucel, M. A., C. M. Aasted, M. P. Petkov, D. Borsook, D. A. Boas and L. Becerra (2015). "Specificity of Hemodynamic Brain Responses to Painful Stimuli: A functional near-infrared spectroscopy study." Sci Rep **5**: 9469.
